# Virological and epidemiological investigations of a severe hand foot and mouth disease outbreak, Vietnam, 2023

**DOI:** 10.1371/journal.pntd.0014789

**Published:** 2026-10-05

**Authors:** Lam Anh Nguyet, Nguyen To Anh, Du Tuan Quy, Nguyen Thi Thu Hong, Ngo Ngoc Quang Minh, Phung Nguyen The Nguyen, Pham Van Quang, Do Duong Kim Han, Nguyen Thi Han Ny, Le Nguyen Truc Nhu, Truong Hoang Chau Truc, Cao Thu Thuy, Nhung Ngoc Hoang, Tran Ba Thien, Le Kim Thanh, Nguyen Thanh Hung, Truong Huu Khanh, Cao Minh Hiep, Nghiem My Ngoc, Nguyen Thanh Dung, Le Nguyen Thanh Nhan, Nguyen Van Vinh Chau, Ha Manh Tuan, H. Rogier van Doorn, Marc Choisy, Tran Tan Thanh, Le Van Tan

**Affiliations:** 1 Oxford University Clinical Research Unit, Ho Chi Minh City, Vietnam; 2 University of Medicine and Pharmacy at Ho Chi Minh City, Ho Chi Minh City, Vietnam; 3 Children’s Hospital 1, Ho Chi Minh City, Vietnam; 4 Pham Ngoc Thach University of Medicine, Ho Chi Minh City, Vietnam; 5 Hospital for Tropical Diseases, Ho Chi Minh City, Vietnam; 6 Department of Health, Ho Chi Minh City, Vietnam; 7 Centre for Tropical Medicine and Global Health, Nuffield Department of Medicine, University of Oxford, Oxford, United Kingdom; Colorado State University, UNITED STATES OF AMERICA

## Abstract

We report on the virological and epidemiological features of a severe hand foot and mouth disease outbreak in Vietnam in 2023. Enterovirus A71 (EV-A71) subgenogroup B5, first emerging around November 2022, and imported into Vietnam through a single introduction, was the primary cause. Convalescent plasma collected from patients infected with subgenogroups B5 in 2014 and 2023 could neutralize both contemporary viruses with comparable titers, suggesting no evidence of immune evasion. However, at one and six months before the start of the outbreak, only 6.7% of 0– < 3 year-old children in Ho Chi Minh City, an epicenter of the outbreak, had neutralizing antibodies against EV-A71. Seroprevalence increased as the outbreak progressed, coinciding with a drop in EV-A71 infected patients residing in Ho Chi Minh City, suggesting that serosurveillance could be a useful tool to assess the risk of pathogen emergence. Our collective findings have provided novel insights into the epidemiological factors driving EV-A71 emergence, and therefore would inform outbreak preparedness and response, and in particular the development of serosurveillance-based approach to assess the risk of pathogen emergence. EV-A71 infection represents an ongoing public health threat, currently affecting countries in the Asia-Pacific region, but with a high potential to become a global problem, and therefore should be closely monitored.

## Author summary

In 2023, Vietnam experienced a large and severe outbreak of hand food and mouth disease in children. Our investigations showed that enterovirus A71 (EV-A71) subgenogroup B5, first emerging around November 2022, and then introduced into Vietnam through a single event, was the main cause. Blood samples from patients who recovered from subgenogroups B5 infection in 2014 and 2023 could neutralize both contemporary viruses without evidence of immune evasion. Before the start of the outbreak, only 6.7% of 0– < 3 year-old children in Ho Chi Minh City, an epicenter of the outbreak, had neutralizing antibodies that can block EV-A71 infection. Seroprevalence increased as the outbreak progressed, while the number of the infected patients decreased. Monitoring population immunity against EV-A71 could help assess the risk of pathogen emergence. EV-A71 infection represents an ongoing public health threat, currently affecting countries in the Asia-Pacific region, but with a high potential to become a global problem, and therefore should be closely monitored.

## Introduction

Over the last decades, enterovirus A-71 (EV-A71) has emerged and become a major public health threat in the Western-Pacific region. Large outbreaks of EV-A71 associated hand foot and mouth disease (HFMD) have occurred across the region, causing millions of hospitalizations in young children [1–6]. Although the infection is mostly self-limited, clinical complications (autonomous dysfunction, pulmonary edema, and brain stem encephalitis) can occur, occasionally leading to fatal outcomes as a consequence [1,7]. Currently, there is no clinically proven effective antivirals to treat the severe patients. Vaccines have successfully been developed in China and Taiwan [8–10], but vaccine deployment has not gone beyond China and Taiwan.

EV-A71 belongs to *Enterovirus* genus of the *Picornaviridae* family. Its genome is single-stranded positive-sense RNA with approximately 7.5 Kbp in length. Based on the viral protein 1 (VP1) coding sequence, EV-A71 is classified into eight genogroups, designated A to H [11]. Genogroups B and C have been circulating worldwide, and are further divided into subgenogroups B1-B5 and C0-C6, respectively [1].

In Vietnam, three large outbreaks of EV-A71 associated HFMD has occurred in 2011 (>200,000 cases, and >200 deaths) [12], in 2018 (>130,000 cases and 17 deaths) [13], and most recently in 2023 (178,000 cases and 32 deaths) [14,15]. Epidemiologically, the reported HFMD cases associated with EV-A71 infection are typically high during the last trimester of an epidemic year when the school year begins. While this pattern was observed during the 2011 and 2018 outbreaks, the 2023 outbreak marked for the first time a prolongation of an EV-A71 outbreak with two peaks in August and November over a period of 8 months (May – December) within a single year [14].

Virologically, subgenogroups C4 and B5 have been replacing each other and been responsible for large HFMD outbreaks in Vietnam over the last decades. Specifically, subgenogroup C4 was responsible for the 2011 and 2018 outbreaks [12,16]. Between 2012 and 2017 during which no outbreak was documented, subgenogroup B5 was predominantly circulating [17,18]. Interestingly, subgenogroup B5 was the main pathogen detected in HFMD patients during the first two months (May and June) of the 2023 outbreaks [14]. Notably, preliminary sequence analysis revealed that EV-A71 circulating during the first two months of the 2023 outbreak represented an emerging virus as it carried a novel glycine residue at position 17 (S17G) within the N-terminus of VP1 [14]. Because VP1 is the most immunogenic protein of EV-A71, the potential effects of this substitution on immune escape of the pathogen warrant further investigation. Likewise, the factors driving the emergence of EV-A71 in 2023 remain unknown. Herein, we aim to address some of those collective existing gaps in knowledge about the virology and epidemiology of the 2023 outbreak to inform future epidemic response, and the development of intervention strategies.

## Materials and methods

### Ethical approval statement

The studies were approved by the Institutional Review Board of Children’s Hospital 1 and the Oxford Tropical Research Ethics Committee (OxTREC) at the University of Oxford, UK. All procedures were performed in accordance with the Declaration of Helsinki. Written informed consent was obtained from each participant and/or their legal guardians.

### Settings

The clinical samples used in the present study were derived from two different studies, a serum bank study, collecting residual samples for serosurveillance, and an observational study of HFMD. Both studies were conducted at Children’s Hospital 1 (CH1) in Ho Chi Minh City (HCMC), Vietnam. CH1 is a tertiary referral hospital for children, including those with HFMD, from HCMC and southern provinces of Vietnam.

### Observational study of HFMD: Patient recruitment and sampling

Patient recruitment and sample collection of the outbreak were conducted between May and December 2023. We included any patient ≤12 years of age admitted to Infectious Diseases Department of CH1 with a clinical diagnosis of HFMD for enrolment in our study. We excluded any patient who the attending physician believed another diagnosis was more likely. We collected information regarding demographics, clinical signs/symptoms, clinical grades, treatments, laboratory tests, length of hospital stay, and outcomes. Additionally, for enterovirus serotype determination, we sampled acute throat and rectal swabs at enrolment, and for immunological analysis, we collected a plasma sample at enrolment and (where appropriate) between 7–14 days after enrollment.

During an outbreak situation, recruitment is naturally biased toward patients with severe diseases, attributed to a large number of severe patients requiring hospitalization.

### HFMD clinical grade classification

According to the Vietnamese Ministry of Health, HFMD is clinically divided into six grades [16], namely Grade 1, 2A, 2B1, 2B2, 3 and 4 – Grade 1: mouth ulcers or vesicles/papules on hands, feet or buttocks, with or without mild fever (<39°C), Grade 2A: central nervous system involvement, (myoclonus reported by parents or caregivers only, fever >39°C or ataxia), Grade 2B1: myoclonus observed by medical staff or history of myoclonus and lethargy or pulse higher than 130 bpm), Grade 2B2: ataxia, cranial nerve palsies, limb weakness, nystagmus, persistent high fever or pulse higher than 150 bpm, Grade 3: autonomic dysfunction with sweating, hypertension, tachycardia and tachypnoea, and Grade 4: cardio-pulmonary compromise with pulmonary oedema or shock syndrome. Patients with Grade 2B1 are considered as having moderately severe HFMD, and those with Grades 2B2, 3 or 4 are considered as having severe HFMD.

### Serum bank and sample selection for serosurveillance

EV-A71 emergence is thought to be in part driven by the accumulation of a sufficient number of susceptible young children in the community. We thus sought to assess if population immunity status could be a useful indicator of disease outbreak, using a serosurveillance system based on hospital residual serum samples that we have recently successfully used to detect the immunity gap prior to a massive measles outbreak in Vietnam in 2024 [19]. Accordingly, we collected from CH1 five samples per 1-year age class from 0 to 15 year-old for each of the 4 months of December 2022 (T1, 6 months before the start of the outbreak), April 2023 (T2), August 2023 (T3) and December 2023 (T4). In total, out of the 300 expected serum samples (4 time points of collection x 15 age classes x 5 samples), 298 were included for analysis as two samples were missing.

### Plasma samples for antigenic analysis

A convenient sample of convalescent plasma collected from HFMD patients with PCR confirmed EV-A71 infection were used. This included 8 samples from patients infected with subgenogroup B5 enrolled into the aforementioned observational study in 2014, and 30 samples from patients infected with the emerging subgenogroup B5 enrolled into the observational study in 2023.

### Laboratory investigations

The collected samples outlined above were analyzed to determine the causative agents, pathogen evolution, immune evasion and population immunity landscape. All analyses were carried out between May 2023 and December 2025.

#### Enterovirus diagnostics and serotype determination.

Enterovirus diagnostics and serotypes/genogroup determination was carried out using a combination of PCR and sequencing approaches as previously described [16,20] (S1 Fig). For cost effectiveness, rectal swabs were prioritized over throat swabs, because emerging evidence suggested that rectal swab PCR provided a higher diagnostic yield [21,22]. In brief, we first extract viral RNA from rectal swabs collected from the study participants, and then utilized a one-step multiplex real-time PCR assay to simultaneously detect enteroviruses in general and EV-A71 specifically in the extracted NA materials. Any specimens positive for enterovirus serotype or EV-A71 were then tested to further identify specific enterovirus serotypes or EV-A71 subgenogroups, respectively, using a combination of VP1 PCR amplification and sequencing of the obtained PCR amplicon [12,23]. Finally, the obtained VP1 sequences were then typed using the online typing tool described in [24]. In case the one-step multiplex real-time PCR analysis of the rectal samples gave a negative result, throat swabs were further analyzed and, if positive, the same workflow was applied to identify enterovirus serotypes or EV-A71 subgenogroups. A confirmed PCR diagnosis was established if the throat or rectal swab was positive by real time PCR.

#### Full VP1 and whole genome sequencing using metagenomics.

EV-A71 positive samples with a Cp of <30 cycles, representing for provinces from where the patients came, were subjected to whole genome amplification and sequencing as previously described [25]. In brief, the selected swabs in viral transport medium were pretreated by a centrifugation step to remove host cells or large cellular components, followed by DNase treatment of the obtained supernatants. Viral nucleic acid (NA) was then isolated using QIAamp viral RNA kit (QIAgen GmbH), and recovered in the elution buffer (provided with the kit). The isolated NA was subjected to cDNA synthesis using Super Script III kit (Invitrogen, Carlsbad, CA, USA) and FR26RV-Endoh primer. The cDNA was then converted to double-stranded DNA using exo-Klenow (Invitrogen), and subsequently pre-amplified using Platinum PCR supermix (Invitrogen) and FR20RV primer. PCR product was then purified and subjected to library preparation using Illumina COVIDseq reagents (Illumina, San Diego, CA, USA), and was finally sequenced using MiSeq reagent kits (Illumina) in an Illumina MiSeq platform (Illumina).

Whole-genome sequence assembly was performed using the Geneious v8.1.5 software package (http://www.geneious.com) utilizing a reference-based mapping approach. This involves the mapping of individual reads of each sample to a reference sequence (Accession numbers: ON502302.1 for subgenogroup C4, LC321989.1 for B5 and MW354740.1 for C1) and manual editing of the consensus. The sequences obtained in this study were submitted to NCBI under accession numbers (accession numbers: PZ583157-PZ583196, PZ583211-PZ583212 and PRJNA1525794).

#### Phylogenetic analysis.

To investigate the relationship between Vietnamese strains and global strains we focused our analysis on complete VP1 sequences submitted to the GenBank as of December 2025 (S1 Table), and those obtained from the present study. The VP1 coding region of EV-A71 have been extensively sequenced, therefore this dataset provided a better geographic representation for the viruses. A time-resolved phylogenetic tree of EV-A71 was reconstructed using Augur v24.2.3 [26], which enabled an accurate estimation of the time of most recent common ancestor (tMRCA) of the pathogen. Accordingly, after removing identical sequences, sequence alignment were carried out using MAFFT, using default settings [27], followed by the inference of a maximum likelihood (ML) tree using the IQ-TREE [28]. Finally, a time- resolved based phylogenetic tree was re-constructed based on the obtained ML tree and information about the sampling dates of the included sequences, using TreeTime [29], which was incorporated into Augur v24.2.3. For all analyses, a subgenogroup C4 sequence (Accession number: MF662693.1) was used as an outgroup.

#### Microneutralisation assay for antigenic analysis and serosurveillance.

For antigenic and serosurveillance analyses, we used an established microneutralization assay that we have previously developed and used for study of EV-A71 infection [30]. In brief, plasma samples were first inactivated at 56°C for 30 min, and were then diluted in serial ratios from 1:8 to a highest dilution that could no longer prevent viral infection of the cell lines. Next, plasma dilutions were incubated with an equal volume of 100 TCID_50_ of the virus at 37^o^C for 1 hour, and were then transferred into a 96-well plate pre-coated with human rabdomyosarcoma cells (RD) (American Type Culture Collection, Manasass, USA). The plate was then incubated in a 5% carbon dioxide incubator at 37^o^C. Cells were observed daily for cytopathic effects (CPE). The antibody titer of the sample was determined by the highest plasma dilution that prevented CPE in 50% of the wells. Each dilution was tested in quadruplicate. Negative and positive controls were included in each 96-well plate.

### Data analysis

For serosurveillance, we divided the study participants into three main age groups, including group 1 (0 – < 3 years), group 2 (3 – < 5 years) and group 3 (5 – < 15 years). Accordingly, the sample size for each time point per group were n = 15 for group 1, n = 10 for group 2, n = 50 for group 3, except T2 of group 3, for which 48 samples were available. For microneutralization analysis, seropositivity was defined as neutralizing antibody titer ≥ 1:8. For quantitative based antigenic analysis, neutralizing antibody titers were log2 transformed.

Data analysis was performed using GraphPad Prism version 9.5.1 (GraphPad Software Inc., Boston, USA), using appropriate statistical tests, including the Chi-square test, Fisher’s exact test, independent T-test, and Mann–Whitney U test, where appropriate.

## Results

### Characteristics of the study participants

Between May and December 2023, we enrolled a total of 360 patients into the observational study. The characteristics of the study participants are summarized in Table 1. The median age was 25.3 months (IQR: 17.2 – 37.2 months). Patients from HCMC accounted for 37.8% (136/360). A total of 286 (79.4%) patients had severe disease, including Grade 2B2 (n = 67), Grade 3 (n = 211) and Grade 4 (n = 8). The duration of hospitalization, median (IQR), was 5 days (4 – 7 days). In terms of outcome at hospital discharge, 345 (96%) patients made a full recovery, and 11 were discharged with an incomplete recovery outcome, with details available in three; one with right limb weakness, one continuing to attend outpatient care, and one continuing to be treated for respiratory symptoms. One was deceased.

**Table 1:** Demographics, clinical grades and outcomes of the study participants.

Characteristics	Total, n = 360	EV-A71, n = 299	Enteroviruses (excluding EV-A71), n = 27	PCR negative, n = 34
**Gender**				
Male, n (%)	214 (59.8)	176 (59.1)	15 (55.6)	23 (69.7)
Female, n (%)	144 (40.2)	122 (40.9)	12 (44.4)	10 (30.3)
**Origin of patients**				
Ho Chi Minh City, n (%)	136 (37.8)	111 (37.1)	9 (33.3)	16 (47.1)
Other provinces/cities, n (%)	224 (62.2)	188 (62.9)	18 (66.7)	18 (52.9)
**Median age in months (IQR)**	25.3 (17.2-37.2)	27.0 (18.5-38.5)	19.7 (13.3-26.6)	16.6 (12.3-21.4)
**Median duration of illness at admission in days (IQR)**	3 (2-4)	3 (2-4)	2 (1-3)	2.5 (1-3)
**Median duration of hospitalisation in days (IQR)**	5 (4-7)	5 (4-7)	6 (4-7)	4.5 (3-7)
**Highest clinical grade over the course of illness, n (%)**				
2A	44 (12.2)	29 (9.7)	4 (14.8)	11 (32.4)
2B1	30 (8.3)	24 (8.0)	2 (7.4)	4 (11.8)
2B2	67 (18.6)	58 (19.4)	4 (14.8)	5 (14.7)
3	211 (58.6)	181 (60.5)	17 (63.0)	13 (38.2)
4	8 (2.2)	7 (2.3)	0	1 (2.9)
**Outcome**				
Complete recovery, n (%)	345 (95.8)	287 (96.0)	25 (92.6)	33 (97.1)
Incomplete recovery*, n (%)	11 (3.1)	8 (2.7)	2 (7.4)	1 (2.9)
Caregiver-requested discharge, n (%)	2 (0.6)	2 (0.7)	0	0
Unknown, n (%)	1 (0.3)	1 (0.3)	0	0
Death, n (%)	1 (0.3)	1 (0.3)	0	0

* Detailed information was obtained from three patients, one attended outpatient care, one had limb weakness, and the other continued treatment for respiratory symptoms. **n = 357.

### Results of molecular investigations

Multiplex RT-PCR analysis revealed an enterovirus infection in 326/360 (90.6%) participants, including 299 (83.1%) were PCR positive for EV-A71, and 27 (7.5%) were PCR positive for enteroviruses but negative for EV-A71 (Fig 1). Of the 299 EV-A71 positive samples, sequencing-based typing analysis was successful in 231/299 (77.3%), including 221 (95.6%) subgenogroup B5, 5 (2.2%) subgenogroup C4, and 5 (2.2%) subgenogroup C1. The remaining 68 swabs that were PCR positive but negative by the molecular typing assays had lower viral loads than those for which typing analysis was successful (S3 Fig).

**Fig 1:**
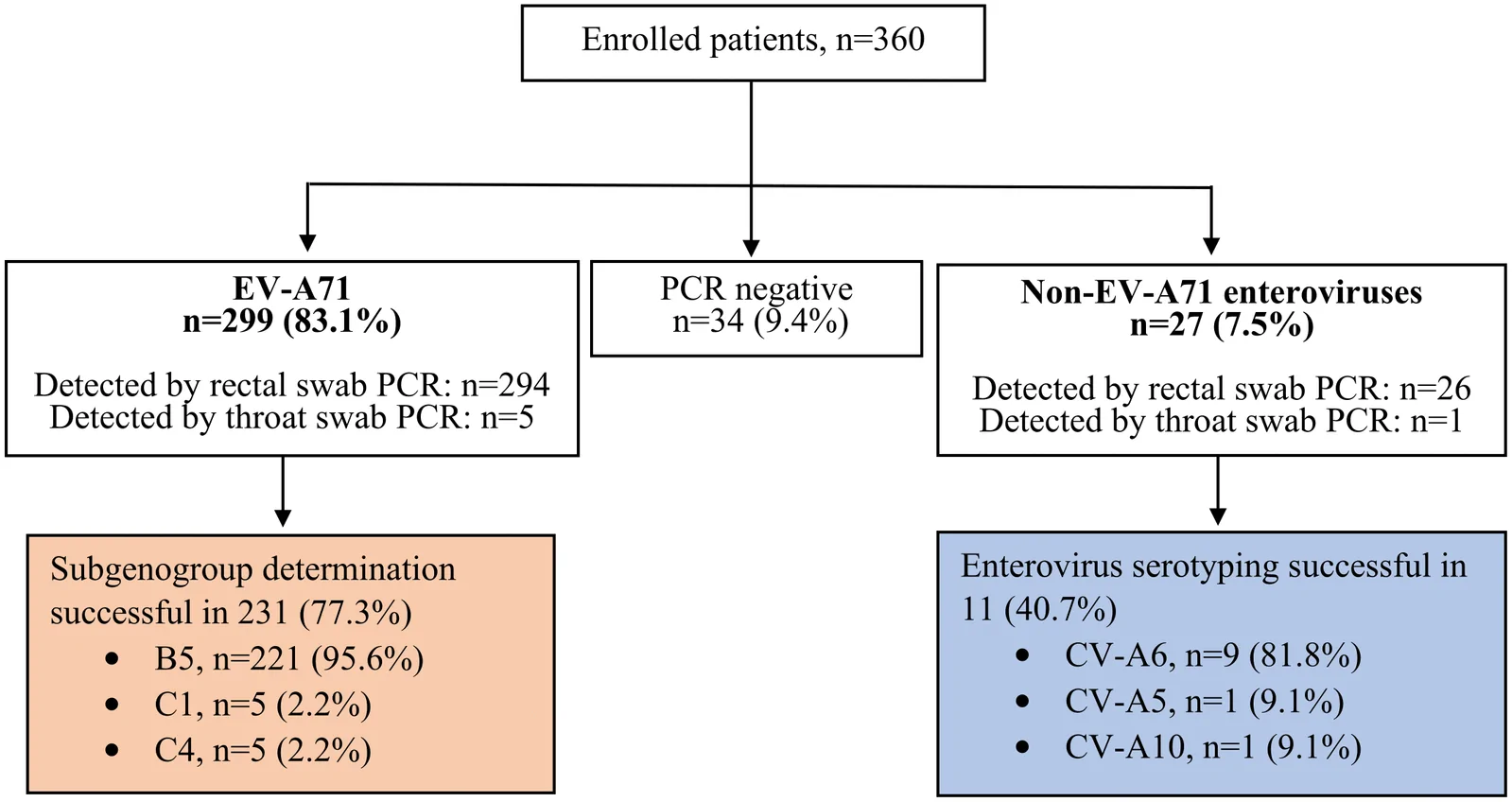
Flowchart illustrating the results of PCR and sequence-based investigation.

Of the 299 EV-A71 infected patients, the proportion of those residing in HCMC was 37.1% (111/299), but decreased over the study period, accounting for 46.1% (105/228) during the first four months (May – August 2023), and 8.5% (6/71) during the last 4 months (September–December 2023) (Fig 2A). Temporally, subgenogroup B5 was detected throughout the study period from May to December 2023, and C4 and C1 were sporadically detected in June, July, August, and November (Fig 2B). Of the 27 enterovirus RT-PCR positive samples but negative for EV-A71 PCR, an enterovirus serotype was successfully in 11 samples, including CVA6, (n = 9, 81.8%), CVA5 (n = 1, 9.1%) and CVA10 (n = 1, 9.1%), (Fig 1).

**Fig 2:**
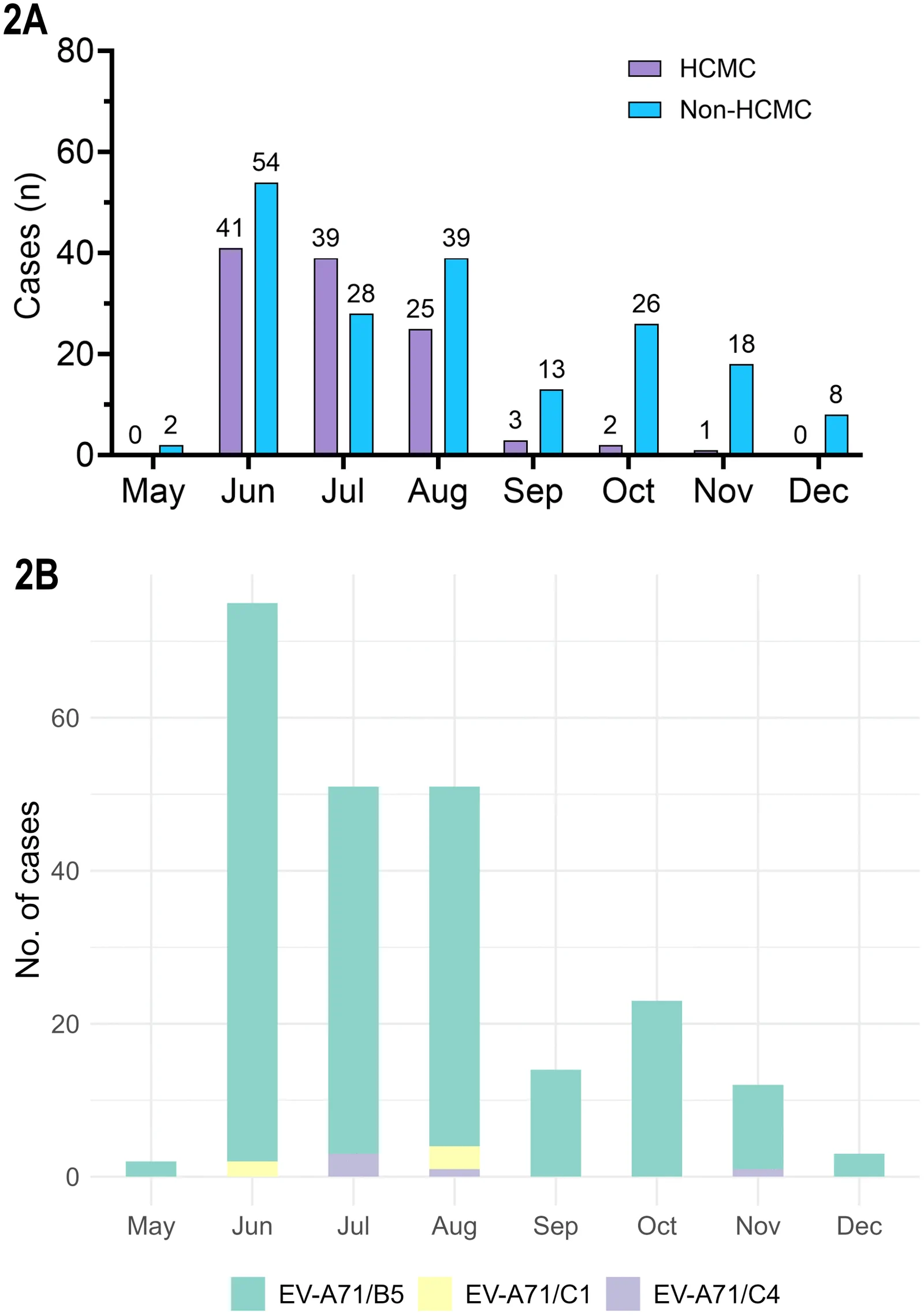
Detection of EV-A71 over the study period, A) Distribution of EV-A71 infected patients residing in HCMC and other provinces (non-HCMC), B) Distribution of EV-A71 subgenogroups between May and June 2023.

The demographics, clinical grades and outcome associated with EV-A71 and enteroviruses infection are summarized in Table 1 and S2 Fig. Of the 286 patients with severe HFMD, EV-A71 infection accounted 86.0% (n = 246), while CVA6 and CVA10 were detected in 7 (2.4%) and 1 (0.3%), respectively. The fatal case had EV-A71 infection but typing effort was not successful.

### Genetic and phylogenetic analyses of dominant EV-A71 subgenogroup B5

As outlined above, we previously described the genetic characteristics of the dominant EV-A71 subgenogroup B5 isolated during the first two months (May and June 2023) of the outbreak. Since the present study revealed subgenogroup B5 remained the dominant virus detected during the last 6 months of the outbreak, we therefore sought to unravel the evolutionary history of this subgenogroup.

A total of 52 full VP1 sequences were obtained using metagenomics-based approach. After excluding identical sequences, 40 unique VP1 sequences were included for analysis, spanning the period from May to December 2023. All except one carried the novel substitution S17G in VP1 that has not been previously reported. Time-scale based phylogenetic analysis revealed that all sequences of the subgenogroup B5 obtained from patients recruited into the study between May and December 2023 formed a single cluster, distinct from subgenogroup B5 strains previously detected in Vietnam and other countries (Fig 3). The estimated tMRCA was around 7 November 2022 (95% confidence interval: 3 June 2022 – 20 February 2023).

**Fig 3:**
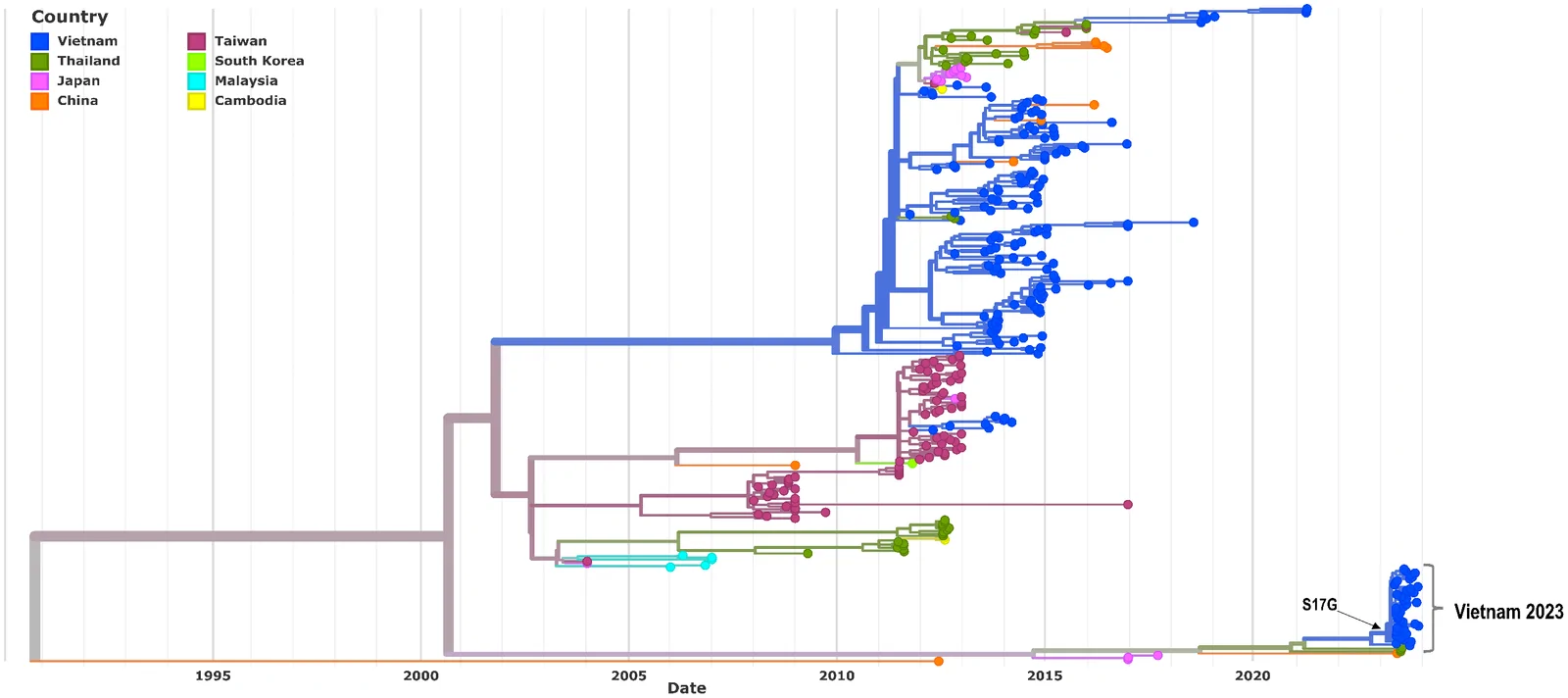
Time-resolved phylogenetic tree depicting the relatedness between emerging EV-A71 subgenogroup B5 and historical strains isolated from Vietnam. Arrow indicates the cluster of the 2023 outbreak.

### Antigenic assessment

All eight convalescent plasma samples collected from subgenogroup B5 infected patients enrolled into the study in 2014 could neutralize the emerging subgenogroup B5 with neutralizing antibody titers comparable with that against the contemporary subgenogroup B5 circulating in Vietnam in 2014, median log2 (IQR): 10 (9 – 10) vs 10 (9.25 – 10), *P* = 0.63 (Fig 4A). Similarly, all 30 convalescent plasma samples collected from patients infected emerging subgenogroup B5 could neutralise both subgenogroups B5 with comparable antibody titers, median log2 (IQR): 10 (9 – 10) vs 10 (9 – 10.25), *P* = 0.36 (Fig 4B).

**Fig 4:**
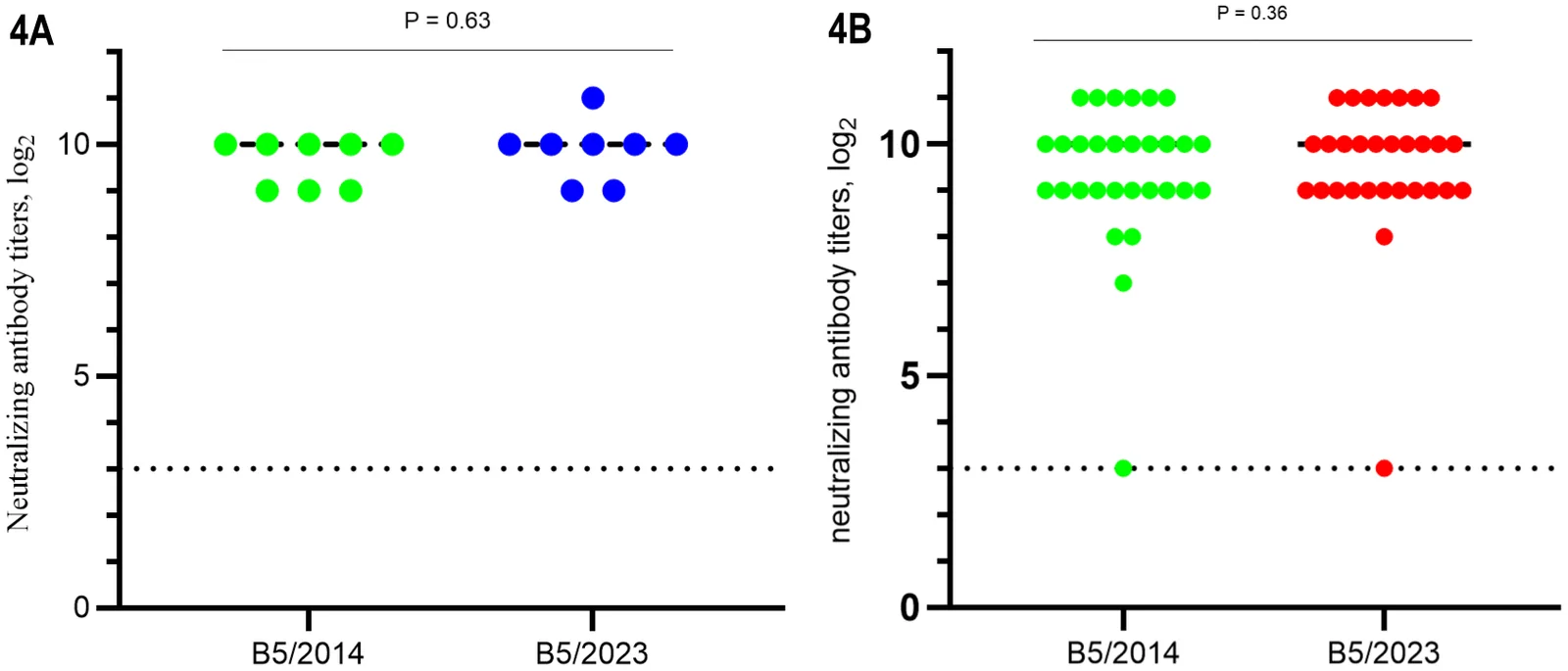
Results of microneutralization analysis assessing the antigenic differences between the 2023 emerging subgenogroup B5 and a subgenogroup B5 isolated in 2023, A) plasma samples collected from patients infected with subgenogroup B5 in 2014, and B) plasma samples collected from patients infected with subgenogroup B5 in 2023.

### Serosurveillance

Of the 300 serum samples collected between December 2022 and December 2023, the proportion of participants with detectable neutralizing antibodies against EV-A71 varied across age groups, and increased over the sampling period (Fig 5A). Specifically, for group 1, this proportion was 6.7% (1/15) in samples collected at both T1 and T2 (95% confidence interval (CI): 0.3%-29.8%), subsequently increasing to 40% (6/15) at T3 (95%CI: 19.8%-69.3%), and 13.3% (2/15) at T4 (95%CI: 2.4%-37.9%). For group 2, the proportion of the tested sera positive for neutralizing antibodies against EV-A71 was 10% (1/10) at T1 (95%CI: 0.5%-40.4%), subsequently increasing to 30% (3/10), 80% (8/10) and 40% (4/10) in samples collected at T2 (95%CI: 10.8%-60.3%), T3 (95%CI: 49%-96.4%) and T4 (95%CI: 16.8%-68.7%), respectively. In those aged 5 – < 15 years (group 3), the seropositivity rate was 50% (25/50) at T1 (95%CI: 36.6%-63.4%), 56.3% (27/48) at T2 (95%CI: 42.3%-69.3%), 52% (26/50) at T3 (95%CI: 38.5%-65.2%), and 62% (31/50) at T4 (95%CI: 48.2%-74.1%).

**Fig 5:**
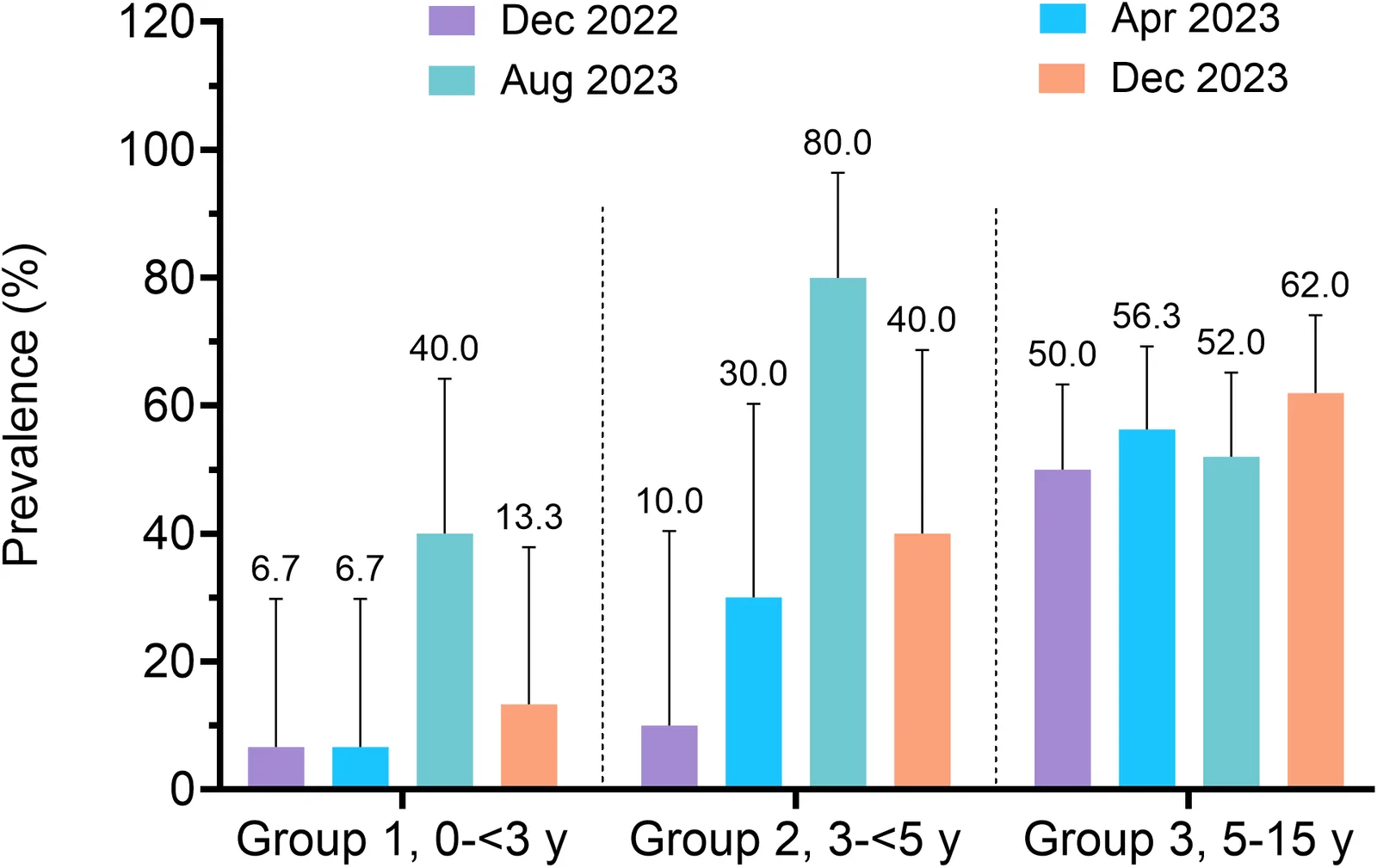
Results of serosurveillance assessing the immune status against EV-A71 in children in Ho Chi Minh City between December 2022 and December 2023.

## Discussion

Here we report on the virological and epidemiological features associated with a large outbreak of severe HFMD in Vietnam in 2023. We showed that emerging EV-A71subgenogroup B5 carrying a novel mutation 17G in the VP1 was the primary cause of the 2023 outbreak, consistent with our initial findings from the first two months of the outbreak [14]. Viruses of EV-A71 subgenogroups C1 and C4 were co-circulating but were sporadically detected alongside CA6 and CVA10. EV-A71 infection was confined to patients with severe disease, especially in those with Grade 3 or 4, while CVA6 and CVA10 were detected in a small proportion of patients with Grade 2B2 or 3. The data therefore reassured that beside EV-A71, CVA (including CVA6, CVA10 and CVA16) can occasionally cause severe diseases, and outbreaks, consistent with previous observations [16] – necessitating the development of multivalent vaccines for HFMD [31].

Time-scale based phylogenetic suggested that the subgenogroup B5 detected during the 2023 outbreak in Vietnam emerged around November 2022. Additionally, all subgenogroup B5 sequences obtained in the present study clustered tightly on the phylogenetic tree, separating them from subgenogroup B5 strains previously isolated from Vietnam and worldwide. This suggests that the emerging subgenogroup B5 of the 2023 outbreak was imported into Vietnam through a single introduction.

Historically, subgenogroup B5 circulation in Vietnam during 2013 and 2017 coincided with a period during which no large outbreak was documented [16,18]. The driving factors that determined the emergence of subgenogroup B5 in 2023 have not been determined. We have started to address this by assessing the immune escape potential of the emerging subgenogroup B5, and the immune landscape among children in HCMC, an epicenter of the outbreak. Using available convalescent plasma collected from HFMD patients infected with two different subgenogroups B5, enrolled in 2014 and 2023, we found no evidence of immune evasion of the 2023 subgenogroup B5 as all the included plasma collected nearly ten years apart could neutralize both subgenogroups with comparable titers. However, results of our serosurveillance study demonstrated that one to six months before the start of the outbreak, only 6.7% of children aged 0 – < 3 years, and 10% of children aged 3 – 5 years in HCMC had neutralizing antibodies against EV-A71 at detectable levels. The population immunity threshold that may trigger an EV-A71 outbreak has not been defined. However, after August 2023, when the seroprevalence reached 40% among 0 – 3-year-old children and 80% among 3 – 5-year-old children, the proportion of EV-A71 infected children from HCMC enrolled into the study during the last four months (September – December) dropped to 8.5% as compared to 46.1% during the first four months (May – August) of the study. Several factors might have contributed to the low seroprevalence observed in our study, but it is likely that the social distancing measures applied during the COVID-19 pandemic represented an important factor, resulting in a large cohort of children susceptible to EV-A71 infection. This explained the affected children of the 2023 outbreak was older than those enrolled into the study during the period before the COVID-19 pandemic [14]. Notably, the impact of COVID-19 pandemic on the transmission of infectious diseases has previously been reported [32,33].

Our study has some limitations. First, during the 2023 outbreak, patient enrolment was biased toward those with more severe disease admitted to the clinical wards. Therefore, the high proportion (79.4%) of the patients with severe HFMD reported in the present study should be interpreted with caution as it may not have fully accounted for the clinical spectrums, or the diversity of enterovirus serotypes, including CVA16, responsible for the infection. Second, as proof of concept, we only focused our serosurveillance in HCMC, an urban setting with a very high population density. While the results highlighted the potential of serosurveillance in assessing the risk of pathogen emergence, future study should be prospectively conducted and be expanded to other provinces to fully account for the utility potential of serosurveillance-based approach in informing HFMD outbreak response. Third, we did not explore potential impacts of the novel amino acid substitution 17G on other phenotypes of EV-A71 (e.g., replication, cell tropism, virulence and transmissibility). Notably, mutations in the N terminus of VP1, where the amino acid 17G residue locates, might increase cell tropism [34]. Alternatively, it may merely represent genetic drift, which merits further research. Fourth, we based our serosurveillance estimates on residual serum samples were collected from a single hospital, the CH1, and therefore our findings may not fully represent community-wide pediatric immunity.

In summary, emerging subgenogroup B5 carrying a novel mutation S17G in the VP1 was the primary cause of the severe HFMD outbreak in Vietnam in 2023. This subgenogroup B5 emerged around November 2022, and was imported into Vietnam via a single introduction. There was however no evidence of immune escape, suggesting that the available vaccines should not be affected. Serosurveillance detected a low level of seroprevalence of EV-A71 in young children at one and six months prior to the start of the outbreak. Our collective findings have provided novel insights into the epidemiological factors driving EV-A71 emergence, and therefore would inform outbreak preparedness and response, and in particular the development of serosurveillance-based approach to assess the risk of pathogen emergence. EV-A71 infection represents an ongoing public health threat, currently affecting countries in the Asia-Pacific region, but with a high potential to become a global concern, and therefore should be closely monitored.

## Supporting information

S1 Fig Flowchart illustrating the laboratory approach to diagnostics and serotyping of HFMD causing enteroviruses. (DOCX)

S2 Fig Enterovirus specific serotypes detected in HFMD patients with different clinical grades. (DOCX)

S3 Fig Distribution of Ct values among swab samples with successful versus unsuccessful enterovirus typing analysis. Higher Ct values are equivalent to lower viral loads. Solid lines represent median values, and dotted lines represent interquartile ranges. Solid lines represent median values, and dotted lines represent interquartile ranges.
(DOCX)

S1 Table Accession numbers of the sequences used for phylogenetic analysis. (DOCX)
