# Supplementary material for: Virological and epidemiological investigations of a severe hand foot and mouth disease outbreak, Vietnam, 2023
Source: PLoS Negl Trop Dis. 2026 Oct 5;20(10):e0014789. doi: 10.1371/journal.pntd.0014789 (PMC13652332; doi:10.1371/journal.pntd.0014789)
Supplement: Supplementary file 1 [file pntd.0014789.s001.docx]

**S1 Fig** : Flowchart illustrating the laboratory approach to diagnostics and serotyping of HFMD causing enteroviruses
