# Supplementary material for: Virological and epidemiological investigations of a severe hand foot and mouth disease outbreak, Vietnam, 2023
Source: PLoS Negl Trop Dis. 2026 Oct 5;20(10):e0014789. doi: 10.1371/journal.pntd.0014789 (PMC13652332; doi:10.1371/journal.pntd.0014789)
Supplement: Supplementary file 3 [file pntd.0014789.s003.docx]

S3 Fig : Distribution of Ct values among swab samples with successful vs unsuccessful enterovirus typing analysis. Higher Ct values are equivalent to lower viral loads. Solid lines represent median values, and dotted lines represent interquartile ranges.
