# Supplementary material for: Virological and epidemiological investigations of a severe hand foot and mouth disease outbreak, Vietnam, 2023
Source: PLoS Negl Trop Dis. 2026 Oct 5;20(10):e0014789. doi: 10.1371/journal.pntd.0014789 (PMC13652332; doi:10.1371/journal.pntd.0014789)
Supplement: Supplementary file 4 [file pntd.0014789.s004.docx]

**S1 Table** : Accession numbers of the sequences used for phylogenetic analysis

| Accession number | Country | Virus |
| --- | --- | --- |
| PV173119.1 | Thailand | EV-A71 B5 |
| PV173120.1 | Thailand | EV-A71 B5 |
| PV173121.1 | Thailand | EV-A71 B5 |
| LC321989.1 | Japan | EV-A71 B5 |
| LC321993.1 | Japan | EV-A71 B5 |
| LC321992.1 | Japan | EV-A71 B5 |
| PP855530.1 | China | EV-A71 B5 |
| PV176308.1 | Taiwan | EV-A71 B5 |
| KT354870.1 | Taiwan | EV-A71 B5 |
| KT354868.1 | Taiwan | EV-A71 B5 |
| MG756708.1 | Taiwan | EV-A71 B5 |
| KF974785.1 | Taiwan | EV-A71 B5 |
| KF974788.1 | Taiwan | EV-A71 B5 |
| KT354866.1 | Taiwan | EV-A71 B5 |
| KT354869.1 | Taiwan | EV-A71 B5 |
| KT354867.1 | Taiwan | EV-A71 B5 |
| KF974780.1 | Taiwan | EV-A71 B5 |
| KF974784.1 | Taiwan | EV-A71 B5 |
| MG756710.1 | Taiwan | EV-A71 B5 |
| KF974779.1 | Taiwan | EV-A71 B5 |
| MG756709.1 | Taiwan | EV-A71 B5 |
| KF974781.1 | Taiwan | EV-A71 B5 |
| KF974783.1 | Taiwan | EV-A71 B5 |
| MG756711.1 | Taiwan | EV-A71 B5 |
| MG756714.1 | Taiwan | EV-A71 B5 |
| MG756707.1 | Taiwan | EV-A71 B5 |
| MG756712.1 | Taiwan | EV-A71 B5 |
| MG756706.1 | Taiwan | EV-A71 B5 |
| HM156065.1 | Taiwan | EV-A71 B5 |
| KF974787.1 | Taiwan | EV-A71 B5 |
| KF974786.1 | Taiwan | EV-A71 B5 |
| FJ357385.1 | Taiwan | EV-A71 B5 |
| MG756713.1 | Taiwan | EV-A71 B5 |
| HM622390.1 | Taiwan | EV-A71 B5 |
| PQ868591.1 | Taiwan | EV-A71 B5 |
| PQ868592.1 | Taiwan | EV-A71 B5 |
| PQ868600.1 | Taiwan | EV-A71 B5 |
| PQ868593.1 | Taiwan | EV-A71 B5 |
| PQ868595.1 | Taiwan | EV-A71 B5 |
| PQ868594.1 | Taiwan | EV-A71 B5 |
| PQ868597.1 | Taiwan | EV-A71 B5 |
| PQ868596.1 | Taiwan | EV-A71 B5 |
| MN629889.1 | South Korea | EV-A71 B5 |
| KF154354.2 | Taiwan | EV-A71 B5 |
| KJ686137.1 | Vietnam | EV-A71 B5 |
| MG756723.1 | Taiwan | EV-A71 B5 |
| MG756721.1 | Taiwan | EV-A71 B5 |
| MG756727.1 | Taiwan | EV-A71 B5 |
| MG756730.1 | Taiwan | EV-A71 B5 |
| MG756754.1 | Taiwan | EV-A71 B5 |
| MG756734.1 | Taiwan | EV-A71 B5 |
| KF974797.1 | Taiwan | EV-A71 B5 |
| MG756753.1 | Taiwan | EV-A71 B5 |
| MG756743.1 | Taiwan | EV-A71 B5 |
| MG756749.1 | Taiwan | EV-A71 B5 |
| MG756747.1 | Taiwan | EV-A71 B5 |
| KF974790.1 | Taiwan | EV-A71 B5 |
| KF134486.1 | Taiwan | EV-A71 B5 |
| MG756742.1 | Taiwan | EV-A71 B5 |
| MG756748.1 | Taiwan | EV-A71 B5 |
| MG756741.1 | Taiwan | EV-A71 B5 |
| LC626900.1 | Japan | EV-A71 B5 |
| KF974794.1 | Taiwan | EV-A71 B5 |
| KF974792.1 | Taiwan | EV-A71 B5 |
| MG756745.1 | Taiwan | EV-A71 B5 |
| MG756732.1 | Taiwan | EV-A71 B5 |
| MG756731.1 | Taiwan | EV-A71 B5 |
| MG756726.1 | Taiwan | EV-A71 B5 |
| KF974793.1 | Taiwan | EV-A71 B5 |
| KF974795.1 | Taiwan | EV-A71 B5 |
| MG756744.1 | Taiwan | EV-A71 B5 |
| MG756738.1 | Taiwan | EV-A71 B5 |
| MG756729.1 | Taiwan | EV-A71 B5 |
| KF974796.1 | Taiwan | EV-A71 B5 |
| MG756733.1 | Taiwan | EV-A71 B5 |
| MG756752.1 | Taiwan | EV-A71 B5 |
| KF154355.1 | Taiwan | EV-A71 B5 |
| MG756739.1 | Taiwan | EV-A71 B5 |
| MG756740.1 | Taiwan | EV-A71 B5 |
| MH716391.1 | Vietnam | EV-A71 B5 |
| KJ686176.1 | Vietnam | EV-A71 B5 |
| MH716390.1 | Vietnam | EV-A71 B5 |
| MH716382.1 | Vietnam | EV-A71 B5 |
| MH716388.1 | Vietnam | EV-A71 B5 |
| MH716387.1 | Vietnam | EV-A71 B5 |
| MH716384.1 | Vietnam | EV-A71 B5 |
| MG756751.1 | Taiwan | EV-A71 B5 |
| MG756750.1 | Taiwan | EV-A71 B5 |
| MG756746.1 | Taiwan | EV-A71 B5 |
| KF974798.1 | Taiwan | EV-A71 B5 |
| JN964686.1 | China | EV-A71 B5 |
| LC375766.1 | Japan | EV-A71 B5 |
| FJ357378.1 | Taiwan | EV-A71 B5 |
| MN053435.1 | Malaysia | EV-A71 B5 |
| MN053432.1 | Malaysia | EV-A71 B5 |
| MN053431.1 | Malaysia | EV-A71 B5 |
| MN053430.1 | Malaysia | EV-A71 B5 |
| KY952186.1 | Thailand | EV-A71 B5 |
| KX372313.1 | Thailand | EV-A71 B5 |
| KU574619.1 | Thailand | EV-A71 B5 |
| KP308430.1 | Cambodia | EV-A71 B5 |
| KP308448.1 | Cambodia | EV-A71 B5 |
| KX372312.1 | Thailand | EV-A71 B5 |
| KX372311.1 | Thailand | EV-A71 B5 |
| KX372310.1 | Thailand | EV-A71 B5 |
| JF738001.1 | Thailand | EV-A71 B5 |
| KX372317.1 | Thailand | EV-A71 B5 |
| KX372318.1 | Thailand | EV-A71 B5 |
| KX372319.1 | Thailand | EV-A71 B5 |
| KR045304.1 | Thailand | EV-A71 B5 |
| KR045291.1 | Thailand | EV-A71 B5 |
| KR045294.1 | Thailand | EV-A71 B5 |
| KR045295.1 | Thailand | EV-A71 B5 |
| KR045297.1 | Thailand | EV-A71 B5 |
| KR045296.1 | Thailand | EV-A71 B5 |
| MN053433.1 | Malaysia | EV-A71 B5 |
| PQ868598.1 | Taiwan | EV-A71 B5 |
| OP947996.1 | Vietnam | EV-A71 B5 |
| MG756695.1 | Taiwan | EV-A71 B5 |
| MG756694.1 | Taiwan | EV-A71 B5 |
| MH716381.1 | Vietnam | EV-A71 B5 |
| MH716261.1 | Vietnam | EV-A71 B5 |
| MH716372.1 | Vietnam | EV-A71 B5 |
| MH716374.1 | Vietnam | EV-A71 B5 |
| MH716373.1 | Vietnam | EV-A71 B5 |
| KJ686222.1 | Vietnam | EV-A71 B5 |
| MH716369.1 | Vietnam | EV-A71 B5 |
| MH716368.1 | Vietnam | EV-A71 B5 |
| MH716370.1 | Vietnam | EV-A71 B5 |
| MH716379.1 | Vietnam | EV-A71 B5 |
| OM417113.1 | Thailand | EV-A71 B5 |
| OM417115.1 | Thailand | EV-A71 B5 |
| KR045302.1 | Thailand | EV-A71 B5 |
| KR045301.1 | Thailand | EV-A71 B5 |
| KX372316.1 | Thailand | EV-A71 B5 |
| KX372315.1 | Thailand | EV-A71 B5 |
| KX372314.1 | Thailand | EV-A71 B5 |
| KX372326.1 | Thailand | EV-A71 B5 |
| KX372323.1 | Thailand | EV-A71 B5 |
| KX372325.1 | Thailand | EV-A71 B5 |
| LC626875.1 | Japan | EV-A71 B5 |
| LC626901.1 | Japan | EV-A71 B5 |
| LC626872.1 | Japan | EV-A71 B5 |
| LC626873.1 | Japan | EV-A71 B5 |
| LC626879.1 | Japan | EV-A71 B5 |
| LC626878.1 | Japan | EV-A71 B5 |
| LC626876.1 | Japan | EV-A71 B5 |
| LC626874.1 | Japan | EV-A71 B5 |
| KR045299.1 | Thailand | EV-A71 B5 |
| KX372327.1 | Thailand | EV-A71 B5 |
| KX372330.1 | Thailand | EV-A71 B5 |
| MH716306.1 | Vietnam | EV-A71 B5 |
| MH716305.1 | Vietnam | EV-A71 B5 |
| MH716302.1 | Vietnam | EV-A71 B5 |
| MH716303.1 | Vietnam | EV-A71 B5 |
| KJ686270.1 | Vietnam | EV-A71 B5 |
| KJ686211.1 | Vietnam | EV-A71 B5 |
| MH716320.1 | Vietnam | EV-A71 B5 |
| MH716344.1 | Vietnam | EV-A71 B5 |
| KJ686140.1 | Vietnam | EV-A71 B5 |
| KJ686302.1 | Vietnam | EV-A71 B5 |
| KJ686277.1 | Vietnam | EV-A71 B5 |
| KJ686296.1 | Vietnam | EV-A71 B5 |
| MH716317.1 | Vietnam | EV-A71 B5 |
| MH716318.1 | Vietnam | EV-A71 B5 |
| MH716316.1 | Vietnam | EV-A71 B5 |
| MH716315.1 | Vietnam | EV-A71 B5 |
| KJ686264.1 | Vietnam | EV-A71 B5 |
| MH716346.1 | Vietnam | EV-A71 B5 |
| KP308454.1 | Cambodia | EV-A71 B5 |
| KJ686128.1 | Vietnam | EV-A71 B5 |
| MH716311.1 | Vietnam | EV-A71 B5 |
| MH716298.1 | Vietnam | EV-A71 B5 |
| MH716299.1 | Vietnam | EV-A71 B5 |
| MH716300.1 | Vietnam | EV-A71 B5 |
| MH716310.1 | Vietnam | EV-A71 B5 |
| MH716301.1 | Vietnam | EV-A71 B5 |
| MH716260.1 | Vietnam | EV-A71 B5 |
| MH716295.1 | Vietnam | EV-A71 B5 |
| MH716294.1 | Vietnam | EV-A71 B5 |
| MH716297.1 | Vietnam | EV-A71 B5 |
| MH716296.1 | Vietnam | EV-A71 B5 |
| KX372331.1 | Thailand | EV-A71 B5 |
| MH716345.1 | Vietnam | EV-A71 B5 |
| MH716309.1 | Vietnam | EV-A71 B5 |
| MH716375.1 | Vietnam | EV-A71 B5 |
| MH716371.1 | Vietnam | EV-A71 B5 |
| MH716313.1 | Vietnam | EV-A71 B5 |
| MH716312.1 | Vietnam | EV-A71 B5 |
| KX430824.1 | Vietnam | EV-A71 B5 |
| MH716267.1 | Vietnam | EV-A71 B5 |
| MH716273.1 | Vietnam | EV-A71 B5 |
| MH716259.1 | Vietnam | EV-A71 B5 |
| MH716272.1 | Vietnam | EV-A71 B5 |
| MH716258.1 | Vietnam | EV-A71 B5 |
| MH716281.1 | Vietnam | EV-A71 B5 |
| MH716283.1 | Vietnam | EV-A71 B5 |
| MH716282.1 | Vietnam | EV-A71 B5 |
| MH716292.1 | Vietnam | EV-A71 B5 |
| MH716287.1 | Vietnam | EV-A71 B5 |
| MH716289.1 | Vietnam | EV-A71 B5 |
| MH716293.1 | Vietnam | EV-A71 B5 |
| MH716284.1 | Vietnam | EV-A71 B5 |
| MH716279.1 | Vietnam | EV-A71 B5 |
| MH716264.1 | Vietnam | EV-A71 B5 |
| MH716277.1 | Vietnam | EV-A71 B5 |
| MH716276.1 | Vietnam | EV-A71 B5 |
| MH716275.1 | Vietnam | EV-A71 B5 |
| LC627083.1 | Vietnam | EV-A71 B5 |
| LC627082.1 | Vietnam | EV-A71 B5 |
| KX372321.1 | Thailand | EV-A71 B5 |
| KX372320.1 | Thailand | EV-A71 B5 |
| KJ686192.1 | Vietnam | EV-A71 B5 |
| MH716262.1 | Vietnam | EV-A71 B5 |
| MH716319.1 | Vietnam | EV-A71 B5 |
| MH716332.1 | Vietnam | EV-A71 B5 |
| MH716333.1 | Vietnam | EV-A71 B5 |
| MH716334.1 | Vietnam | EV-A71 B5 |
| MH716329.1 | Vietnam | EV-A71 B5 |
| MH716324.1 | Vietnam | EV-A71 B5 |
| MH716327.1 | Vietnam | EV-A71 B5 |
| MH716328.1 | Vietnam | EV-A71 B5 |
| MH716331.1 | Vietnam | EV-A71 B5 |
| MH716330.1 | Vietnam | EV-A71 B5 |
| MH716326.1 | Vietnam | EV-A71 B5 |
| MH716325.1 | Vietnam | EV-A71 B5 |
| KJ686234.1 | Vietnam | EV-A71 B5 |
| MH716323.1 | Vietnam | EV-A71 B5 |
| KJ686297.1 | Vietnam | EV-A71 B5 |
| MH716322.1 | Vietnam | EV-A71 B5 |
| MH716321.1 | Vietnam | EV-A71 B5 |
| MH716338.1 | Vietnam | EV-A71 B5 |
| MH716337.1 | Vietnam | EV-A71 B5 |
| MH716336.1 | Vietnam | EV-A71 B5 |
| MH716335.1 | Vietnam | EV-A71 B5 |
| MH716308.1 | Vietnam | EV-A71 B5 |
| MH716307.1 | Vietnam | EV-A71 B5 |
| KR045300.1 | Thailand | EV-A71 B5 |
| KX372329.1 | Thailand | EV-A71 B5 |
| MH716378.1 | Vietnam | EV-A71 B5 |
| MH716377.1 | Vietnam | EV-A71 B5 |
| MH716340.1 | Vietnam | EV-A71 B5 |
| MH716339.1 | Vietnam | EV-A71 B5 |
| MH716341.1 | Vietnam | EV-A71 B5 |
| LC627068.1 | Vietnam | EV-A71 B5 |
| MH716263.1 | Vietnam | EV-A71 B5 |
| MH716364.1 | Vietnam | EV-A71 B5 |
| MH716353.1 | Vietnam | EV-A71 B5 |
| MH716350.1 | Vietnam | EV-A71 B5 |
| MH716352.1 | Vietnam | EV-A71 B5 |
| MH716351.1 | Vietnam | EV-A71 B5 |
| MH716348.1 | Vietnam | EV-A71 B5 |
| MH716347.1 | Vietnam | EV-A71 B5 |
| MH716360.1 | Vietnam | EV-A71 B5 |
| MH716361.1 | Vietnam | EV-A71 B5 |
| MH716363.1 | Vietnam | EV-A71 B5 |
| MH716362.1 | Vietnam | EV-A71 B5 |
| MH716354.1 | Vietnam | EV-A71 B5 |
| MH716355.1 | Vietnam | EV-A71 B5 |
| MH716356.1 | Vietnam | EV-A71 B5 |
| MH716358.1 | Vietnam | EV-A71 B5 |
| MH716357.1 | Vietnam | EV-A71 B5 |
| MK344771.1 | China | EV-A71 B5 |
| LC627067.1 | Vietnam | EV-A71 B5 |
| LC627075.1 | Vietnam | EV-A71 B5 |
| LC627076.1 | Vietnam | EV-A71 B5 |
| LC627071.1 | Vietnam | EV-A71 B5 |
| LC627077.1 | Vietnam | EV-A71 B5 |
| LC627073.1 | Vietnam | EV-A71 B5 |
| LC627074.1 | Vietnam | EV-A71 B5 |
| LC627072.1 | Vietnam | EV-A71 B5 |
| MH716365.1 | Vietnam | EV-A71 B5 |
| LC627080.1 | Vietnam | EV-A71 B5 |
| MN966512.1 | China | EV-A71 B5 |
| MH716359.1 | Vietnam | EV-A71 B5 |
| LC627084.1 | Vietnam | EV-A71 B5 |
| KU647000.1 | China | EV-A71 B5 |
| LC627081.1 | Vietnam | EV-A71 B5 |
| LC627079.1 | Vietnam | EV-A71 B5 |
| MH716367.1 | Vietnam | EV-A71 B5 |
| OP947997.1 | Vietnam | EV-A71 B5 |
| LC627078.1 | Vietnam | EV-A71 B5 |
| MN966518.1 | China | EV-A71 B5 |
| MN966516.1 | China | EV-A71 B5 |
| MN966514.1 | China | EV-A71 B5 |
| MN966513.1 | China | EV-A71 B5 |
| MF662693.1 | China | EV-A71 C4 |
