# Supplementary material for: Virological and epidemiological investigations of a severe hand foot and mouth disease outbreak, Vietnam, 2023
Source: PLoS Negl Trop Dis. 2026 Oct 5;20(10):e0014789. doi: 10.1371/journal.pntd.0014789 (PMC13652332; doi:10.1371/journal.pntd.0014789)
Supplement: Supplementary file 5 [file pntd.0014789.s006.docx]

PNTD-D-26-01095
Virological and epidemiological investigations of a severe hand foot and mouth disease outbreak, Vietnam, 2023

**Additional Editor Comments (if provided):**

Your manuscript has been reviewed by 3 experts, each of whom has provided comments that need to be addressed. Please review the comments, edit your manuscript in response, and resubmit along with a point-by-point response to the reviewer comments and requests. Thank you.


**Journal Requirements:**

If the reviewer comments include a recommendation to cite specific previously published works, please review and evaluate these publications to determine whether they are relevant and should be cited. There is no requirement to cite these works unless the editor has indicated otherwise.

**Comment** 1) Please ensure that the CRediT author contributions listed for every co-author are completed accurately and in full.
At this stage, the following Authors/Authors require contributions: Lam Anh Nguyet, Nguyen To Anh, Du Tuan Quy, Nguyen Thi Thu Hong, Ngo Ngoc Quang Minh, Phung Nguyen The Nguyen, Pham Van Quang, Do Duong Kim Han, Nguyen Thi Han Ny, Le Nguyen Truc Nhu, Truong Hoang Chau Truc, Cao Thu Thuy, Nhung Ngoc Hoang, Tran Ba Thien, Le Kim Thanh, Nguyen Thanh Hung, Truong Huu Khanh, Cao Minh Hiep, Nghiem My Ngoc, Nguyen Thanh Dung, Le Nguyen Thanh Nhan, Nguyen Van Vinh Chau, Ha Manh Tuan, H. Rogier van Doorn, Marc Choisy, Tran Tan Thanh, and Le Van Tan. Please ensure that the full contributions of each author are acknowledged in the "Add/Edit/Remove Authors" section of our submission form.
The list of CRediT author contributions may be found here: [https://journals.plos.org/plosntds/s/authorship#loc-author-contributions](https://journals.plos.org/plosntds/s/authorship#loc-author-contributions)

**Response**: We have now revised the author contributions section as commented.

**Comment** 2) Please provide an Author Summary. This should appear in your manuscript between the Abstract (if applicable) and the Introduction, and should be 150-200 words long. The aim should be to make your findings accessible to a wide audience that includes both scientists and non-scientists. Sample summaries can be found on our website under Submission Guidelines:
[https://journals.plos.org/plosntds/s/submission-guidelines#loc-parts-of-a-submission](https://journals.plos.org/plosntds/s/submission-guidelines#loc-parts-of-a-submission)

**Response**: Done

**Comment** 3) Please upload all main figures as separate Figure files in .tif or .eps format. For more information about how to convert and format your figure files please see our guidelines: 
<https://journals.plos.org/plosntds/s/figures>

**Response**: Done

**Comment** 4) Please upload a copy of Figure Fig 1-5 which you refer to in your text on pages 10, 11, and 12. Or, if the figure is no longer to be included as part of the submission please remove all reference to it within the text.

**Response**: Done

**Comment** 5) We have noticed that you have uploaded Supporting Information files, but you have not included a list of legends. Please add a full list of legends for your Supporting Information files after the references list.

**Response**: Done.

**Comment** 6) Please amend your detailed Financial Disclosure statement. This is published with the article. It must therefore be completed in full sentences and contain the exact wording you wish to be published.
i) State the initials, alongside each funding source, of each author to receive each grant. For example: "This work was supported by the National Institutes of Health (####### to AM; ###### to CJ) and the National Science Foundation (###### to AM)."
ii) State what role the funders took in the study. If the funders had no role in your study, please state: "The funders had no role in study design, data collection and analysis, decision to publish, or preparation of the manuscript."
iii) If any authors received a salary from any of your funders, please state which authors and which funders..
If you did not receive any funding for this study, please simply state: u201cThe authors received no specific funding for this work.u201d

**Response**: Done

**Comment** 7) We have amended your Competing Interest statement to comply with journal style. We kindly ask that you double check the statement and let us know if anything is incorrect.

**Response**: Looking good. Thank you.

**Comment** 8) Thank you for uploading your study's underlying data set. Unfortunately, the repository you have noted in your Data Availability statement does not qualify as an acceptable data repository according to PLOS's standards.

At this time, please upload the minimal data set necessary to replicate your study's findings to a stable, public repository (such as figshare or Dryad) and provide us with the relevant URLs, DOIs, or accession numbers that may be used to access these data. For a list of recommended repositories and additional information on PLOS standards for data deposition, please see 

[https://journals.plos.org/plosntds/s/recommended-repositories](https://apc01.safelinks.protection.outlook.com/?url=https%3A%2F%2Ftrack.editorialmanager.com%2FCL0%2Fhttps%3A%252F%252Fjournals.plos.org%252Fplosntds%252Fs%252Frecommended-repositories%2F1%2F010f01a03c358085-e7f5694d-f752-4edb-80ae-48d08eb52e43-000000%2FcMJAMh3T4OScEl82jzrfnmrDTXpgLKbEwneKhmLdRwQ%3D258&data=05%7C02%7Ctanlv%40oucru.org%7C919854e54d3b4edf95f008df0325e329%7C22514748bbda4cb89795bfa3fbeec018%7C0%7C0%7C639233133435757515%7CUnknown%7CTWFpbGZsb3d8eyJFbXB0eU1hcGkiOnRydWUsIlYiOiIwLjAuMDAwMCIsIlAiOiJXaW4zMiIsIkFOIjoiTWFpbCIsIldUIjoyfQ%3D%3D%7C0%7C%7C%7C&sdata=zBXgAXhxrUeQvTJMspaNR5kEXDQkX2wlWc7PFtODNwI%3D&reserved=0)

**Response**: We have now submitted reported dataset necessary to replicate our findings to figshare, https://doi.org/10.6084/m9.figshare.33459943

**Reviewers' Comments:**

**Reviewer #1**: It's a nice piece combining molecular epidemiology, phylogenetics, antigenic characterization and serosurveillance around an important outbreak, and the central narrative (single introduction of an antigenically unchanged B5 lineage into an immunologically naïve population) is compelling. However, there are several numerical inconsistencies between the text, figures, and legends that need to be resolved, plus some clarity and statistical issues.

**Comment** 1.Figure 4 legend appears to have the wrong year. The legend states the comparison is between "the 2023 emerging subgenogroup B5 and a subgenogroup B5 isolated in 2024," but the entire text, panel labels (B5/2014 vs B5/2023), and Methods (plasma from patients infected in 2014 and 2023) all describe a 2014-vs-2023 comparison, not 2024. Please correct "2024" to "2014" in the Figure 4 legend to avoid confusing readers about what is actually being compared.

**Response**: We have now corrected this typo, changing 2024 to 2023 as commented.

**Comment** 2.Figure 5 x-axis/legend says "T4, Dec 2024," but the Methods and figure caption text state T4 = December 2023. This is a second instance of an apparent 2023/2024 mix-up and directly affects interpretation of the serosurveillance timeline, which is central to the paper's argument. Please correct the T4 label to "Dec 2023" in Figure 5 and double-check that no other 2023/2024 date errors exist elsewhere in the manuscript.

**Response**: Please accept our apologies for the confusions caused by those typos. We have now correct the typo, changing from Dec 2024 to Dec 2023 as commented.

**Comment** 3.Minor percentage discrepancy for subgenogroup typing success. The Results text states subgenogroup determination was "successful in 231/299 (77.5%)," while Figure 1 reports 77.3% for the same numerator/denominator. 231/299 = 77.3%, so the text figure appears to be the error. Please correct "77.5%" to "77.3%" in the Results text to match Figure 1 and the underlying calculation.

**Response**: we have now corrected the above typo as commented.

**Comment** 4.Genogroup nomenclature inconsistency in the Introduction. The text states EV-A71 "is classified into eight genogroups, designated A to F," but A through F comprises only six letters/groups. Please verify the correct number of genogroups against the cited literature and correct either the number ("eight") or the letter range to be internally consistent.

**Response**: We have not edited the text to reflect that there have been 8 genogroups described in the literature, and cited a paper to support our statement (PMID: 30377626).

**Comment** 5.Very small sample sizes underlying key seroprevalence estimates, without confidence intervals. Several headline percentages are based on extremely small denominators (e.g., 1/15, 1/10, 6/15), yet are reported only as point estimates (6.7%, 10%, 40%, etc.) without any measure of uncertainty. Given that a central claim of the paper is that seroprevalence "increased" over time and this is used to support a serosurveillance-based early-warning concept, the precision of these estimates matters a great deal. Please report 95% confidence intervals (e.g., Wilson or Clopper-Pearson) alongside all seroprevalence point estimates, and discuss the resulting uncertainty explicitly, especially for group 1 and group 2 where n is smallest.

**Response:** 95% confidence intervals have now been calculated and shown in Figure 5, where the results of serosurveillance were visualized. Accordingly, we have edited the related text by adding these new findings to the ms, line: 289-298 reads “Specifically, for group 1, this proportion was 6.7% (1/15) in samples collected at both T1 and T2 (95% confidence interval (CI): 0.3%-29.8%), subsequently increasing to 40% (6/15) at T3 (95%CI: 19.8%-69.3%), and 13.3% (2/15) at T4 (95%CI: 2.4%-37.9%). For group 2, the proportion of the tested sera positive for neutralizing antibodies against EV-A71 was 10% (1/10) at T1 (95%CI: 0.5%-40.4%), subsequently increasing to 30% (3/10), 80% (8/10) and 40% (4/10) in samples collected at T2 (95%CI: 10.8%-60.3%), T3 (95%CI: 49%-96.4%) and T4 (95%CI: 16.8%-68.7%), respectively. In those aged 5 – <15 years (group 3), the seropositivity rate was 50% (25/50) at T1 (95%CI: 36.6%-63.4%), 56.3% (27/48) at T2 (95%CI: 42.3%-69.3%), 52% (26/50) at T3 (95%CI: 38.5%-65.2%), and 62% (31/50) at T4 (95%CI: 48.2%-74.1%).”

**Comment** 6.Reference section header reads "REFEENCES", a typo for "REFERENCES." Please correct this heading.

**Comment** 7.Abstract: "0–<3 year-cold children" should read "year-old." Correct typo.

**Comment** 8.Methods (Data analysis section): "particiapnts" should read "participants." Correct typo.

**Comment** 9.Results: "with details availabe in three" should read "available." Correct typo.

**Response**: The above typoes mentioned under comments 6-9 have now been corrected. Thank you.

**Reviewer #2**: The authors have conducted a comprehensive investigation into the 2023 EV-A71 outbreak in Vietnam, integrating molecular epidemiology, serology, and antigenic analysis. The study carries clear public health relevance. However, several points require further clarification and revision before the manuscript can be accepted.

1. Specific comments:
**Comment** (1) In the Figure 4 legend, the authors refer to "a subgenogroup B5 isolated in 2024." Given that the study timeline spans 2022–2023, this date appears inconsistent and should be corrected to align with the actual isolation year.

**Response**: We have now corrected this typo.

**Comment** (2) Similarly, the legend of Figure 5 contains an error indicating "Dec 2024," while the study period is defined as December 2022 to December 2023. This should be amended accordingly.

**Response**: We have now corrected this typo, changing from 2024 to 2023.

**Comment** (3) The Results section reports that 360 patients were enrolled, of whom 299 tested positive for EV-A71, and subgenogroup data were available for 231 cases. The authors should clarify why the remaining 68 positive cases could not be subtyped and provide a more detailed discussion of this discrepancy.

**Response**: We have now elaborated more on this, line: 228-230 reads “The remaining 68 swabs that were PCR positive but negative by the molecular typing assays had lower viral loads than those for which typing analysis was successful (Figure S3).”

**Comment** (4) In the "Whole-genome sequence assembly" section, the authors describe using a reference-based mapping approach but do not specify the reference genome used. Please include the corresponding GenBank accession number(s) in this section.

**Response**: We have now added the GenBank Accession Numbers of the reference sequences used to the ms, line: 166-169 reads “This involves the mapping of individual reads of each sample to a reference sequence (Accession numbers: ON502302.1 for subgenogroup C4, LC321989.1 for B5 and MW354740.1 for C1) and manual editing of the consensus.”

**Comment** (5) In Line 152, Geneious 8.1.should be Geneious v8.1.5.

**Response**: Done

**Comment** (6) In the Data Availability section, I would recommend providing the NCBI SRA accession ID(s) rather than only the assembled sequences, to allow full transparency and reproducibility.

**Response**: We have submitted the raw sequences to the NCBI SRA and are still waiting for the assigned Accession Numbers. In the meantime, the GenBank Accession Numbers of the assembled sequences have now been released and added to the ms.

2. Questions on scientific logic and discussion

**Comment** (1) The S17G mutation in VP1 N-terminus was shown not to affect immune escape, but its biological significance remains unclear. The authors should discuss whether this change might influence viral fitness or cell tropism, or whether it simply represents genetic drift.

**Response**: We have now revised the associated text to capture the areas that the reviewer suggested, line: 348-352 reads “Third, we did not explore potential impacts of the novel amino acid substitution 17G on other phenotypes of EV-A71 (e.g., replication, cell tropism, virulence and transmissibility). Notably, mutations in the N terminus of VP1, where the amino acid 17G residue locates, might increase cell tropism [25]. Alternatively, it may merely represent genetic drift, which merits further research.”

**Comment** (2) Serum samples were collected from hospital inpatients and may not fully represent community-wide pediatric immunity. This limitation should be acknowledged, along with its potential impact on population-level seroprevalence estimates.

**Response**: We have now acknowledge the above mentioned limitation to the discussion, line: 352-354 reads “Fourth, we based our serosurveillance estimates on residual serum samples collected from a single hospital, the CH1, and therefore our findings may not fully represent community-wide pediatric immunity.”

**Comment** (3) The proportion of severe cases (79.4%) is strikingly high. While hospital-based surveillance partly explains this, the authors should discuss whether mild cases in the community were systematically undercounted, and exercise caution when generalizing these findings to the broader outbreak profile.

**Response**: We have discussed this as the first limitation of our study and have now add the figure (79.4%) to the associated text and elaborate more on this to strengthen our discussion, line: 339-343 reads “First, during the 2023 outbreak, patient enrolment was biased toward those with more severe disease admitted to the clinical wards. Therefore, the high proportion (79.4%) of the patients with severe HFMD reported in the present study should be interpreted with caution as it may not have fully accounted for the clinical spectrums, or the diversity of enterovirus serotypes, including CVA16, responsible for the infection.”

**Comment**: Overall, the study is of interest, but I would recommend a minor revision to address the above issues.

**Response**: Thank you. We have now revised our ms as commented.

**Reviewer #3**: This is a well-conducted study investigating the virological and epidemiological characteristics of the severe HFMD outbreak in Vietnam in 2023. Overall, the methodology is appropriate and sufficiently described. The study appears to have been conducted systematically, and the limitations have been adequately addressed in the Discussion.

However, there are several minor comments and queries that should be addressed to improve the clarity and accuracy of the manuscript:

1. Introduction

**Comment**: The authors state:

“Over the last decades, enterovirus A-71 (EV-A71) has emerged and become a major public health threat in the Western-Pacific region. Large outbreaks of EV-A71-associated hand, foot and mouth disease (HFMD) have occurred across the region, causing millions of hospitalizations in young children [1, 2].”

One of the references cited is relatively old (1998), while the other is a review article. The authors should reconsider the references used to support this statement and, where possible, include more recent epidemiological studies or surveillance data to provide up-to-date evidence for the continuing public health significance of EV-A71 in the Western-Pacific region.

**Response**: We have now cited 4 additional papers (references 3-6), published over the last 2 years.

**Comment**: 2. Introduction

The authors state:

“…and most recently in 2023 (>180,000 cases and 31 deaths) [9].”

However, Reference 9 appears to report 7 deaths, rather than 31 deaths. Please verify this information and revise the statement accordingly. If the figure of 31 deaths is supported by another reliable source, the authors should provide an appropriate reference.

**Response**: We have now cited the updated Figures (178,000 cases and 32 deaths) reported by the Vietnamese Ministry of Health and cite the MOH report as a source of information.

**Comment**: 3. Materials and Methods – Serum Bank and Sample Selection for Serosurveillance

The description of the serum sample selection is unclear:

“4 time points of collection × 15 age classes × 5 samples”

It is not clear whether the study included 15 age classes with 5 samples collected at each of 4 time points, or 16 age (0 to 15 age) classes with 4 samples per age class. Please clarify the sampling scheme and provide the total number of serum samples included in the serosurveillance analysis.

**Response**: Please accept our apologies for the confusion and allow us to clarify on this. The term “per 1-year age class from 0 to 15 year-old” meant 15 age classes in total as age classes were defined as 0-1, >1-2, >2-3, and so on.

**Comment** 4. Materials and Methods – Enterovirus Diagnostics and Serotype Determination

The authors state that viral RNA was extracted from rectal swabs collected from study participants. Please provide a justification for using rectal swabs rather than throat swabs for viral RNA detection.

Previous work has suggested that throat swabs may provide better diagnostic sensitivity than rectal swabs for EV-A71 detection. For example:

Ooi EE, et al. Evaluation of Different Clinical Sample Types in Diagnosis of Human Enterovirus 71-Associated Hand-Foot-and-Mouth Disease. Journal of Clinical Microbiology. 2007;45(6). [https://doi.org/10.1128/JCM.01394-06](https://doi.org/10.1128/JCM.01394-06).

Please discuss the rationale for the choice of specimen type and whether the use of rectal swabs could have influenced the detection rate of EV-A71 or other enteroviruses.

In addition, it is notable that CV-A16 was not detected in this study. Please provide some discussion regarding this observation. For example, could this finding be related to the sampling population, specimen type, circulating enterovirus epidemiology, or the timing of sample collection?

**Response**: We would like to thank the reviewer for their comment and the JCM paper. We based our testing strategies on the emering evidence, demonstrating that rectal swabs were provided a higher diagnostic yield. Accordingly, we have cited the recent papers in support of our testing strategies, line: 139-141, reads “For cost effectiveness, rectal swabs were prioritized over throat swabs because, emerging evidence suggested that rectal swab PCR provided a higher diagnostic yield (PMIDs: 29688329 and 36371008)”.

We feel that prioritising rectal swabs over throat swabs would not affect our final results, as throat swabs would be utimately tested in patients whose rectal swabs were PCR negative. However, we agree with the reviewer that it was likely the study population or the sampling approach was the contributing factor. We have therefore revised the associated text to reflect on this, line: 340-343 reads “Therefore, the high proportion (79.4%) of the patients with severe HFMD reported in the present study should be interpreted with caution as it may not have fully accounted for the clinical spectrums, or the diversity of enterovirus serotypes, including CVA16, responsible for the infection.”

**Comment**: 5. Discussion

The authors conclude:

“EV-A71 infection represents an ongoing public health threat, currently affecting countries in the Asia-Pacific region, but with a high potential to become a global concern, and therefore should be closely monitored.”

The authors may wish to broaden this statement to acknowledge that other enteroviruses associated with HFMD, particularly CV-A6 and CV-A10, are also increasingly important from a public health perspective. These viruses have been associated with outbreaks and, in some settings, severe or atypical clinical manifestations.

Therefore, the authors could consider emphasizing the importance of continued surveillance of EV-A71 as well as other HFMD-associated enteroviruses, rather than focusing exclusively on EV-A71. This would provide a broader perspective on the evolving epidemiology and public health significance of HFMD.

**Response**: We have now revised the associated text to further emphasise the pubic health importance of CVA, line 306-309 reads “The data therefore reassured that beside EV-A71, CVA (including CVA6, CVA10 and CVA16) can occasionally cause severe diseases, and outbreaks, consistent with previous observations [10] – necessitating the development of multivalent vaccines for HFMD [22].”

**Figure resubmission:**

While revising your submission, we strongly recommend that you use PLOS’s NAAS tool ([https://ngplosjournals.pagemajik.ai/artanalysis](https://ngplosjournals.pagemajik.ai/artanalysis)) to test your figure files. NAAS can convert your figure files to the TIFF file type and meet basic requirements (such as print size, resolution), or provide you with a report on issues that do not meet our requirements and that NAAS cannot fix.

After uploading your figures to PLOS’s NAAS tool - [https://ngplosjournals.pagemajik.ai/artanalysis](https://ngplosjournals.pagemajik.ai/artanalysis), NAAS will process the files provided and display the results in the "Uploaded Files" section of the page as the processing is complete. If the uploaded figures meet our requirements (or NAAS is able to fix the files to meet our requirements), the figure will be marked as "fixed" above. If NAAS is unable to fix the files, a red "failed" label will appear above. When NAAS has confirmed that the figure files meet our requirements, please download the file via the download option, and include these NAAS processed figure files when submitting your revised manuscript.

**Response**: We have submitted the required Figures with the required resolutions as requested.

**Reproducibility:**

To enhance the reproducibility of your results, we recommend that authors of applicable studies deposit laboratory protocols in protocols.io, where a protocol can be assigned its own identifier (DOI) such that it can be cited independently in the future. Additionally, PLOS ONE offers an option to publish peer-reviewed clinical study protocols. Read more information on sharing protocols at [https://plos.org/protocols?utm_medium=editorial-email&utm_source=authorletters&utm_campaign=protocols](https://plos.org/protocols?utm_medium=editorial-email&utm_source=authorletters&utm_campaign=protocols)

**Response**: Thank you for the opportunity. Methods have been detailed in the ms and have previously been published (references: 20, 25 and 30) and we will consider our study protocol for future submission after discussing internally.
