# Supplementary material for: Virological and epidemiological investigations of a severe hand foot and mouth disease outbreak, Vietnam, 2023
Source: PLoS Negl Trop Dis. 2026 Oct 5;20(10):e0014789. doi: 10.1371/journal.pntd.0014789 (PMC13652332; doi:10.1371/journal.pntd.0014789)
Supplement: Peer review history

# Peer review: Decision Letter 0

**DOI:** 10.1371/journal.pntd.0014789.r001

**Authors:** Richard Bowen, Sujatha Sunil

25 Aug 2026

PNTD-D-26-01095

Virological and epidemiological investigations of a severe hand foot and mouth disease outbreak, Vietnam, 2023

PLOS Neglected Tropical Diseases

Dear Dr. Tan,

Thank you for submitting your manuscript to PLOS Neglected Tropical Diseases. After careful consideration, we feel that it has merit but does not fully meet PLOS Neglected Tropical Diseases's publication criteria as it currently stands. Therefore, we invite you to submit a revised version of the manuscript that addresses the points raised during the review process.

Please submit your revised manuscript within by Oct 24 2026 11:59PM. If you will need more time than this to complete your revisions, please reply to this message or contact the journal office at plosntds@plos.org. When you're ready to submit your revision, log on to https://www.editorialmanager.com/pntd/ and select the 'Submissions Needing Revision' folder to locate your manuscript file.

Please include the following items when submitting your revised manuscript:

* A letter that responds to each point raised by the editor and reviewer(s). You should upload this letter as a separate file labeled 'Response to Reviewers'. This file does not need to include responses to any formatting updates and technical items listed in the 'Journal Requirements' section below.

* A marked-up copy of your manuscript that highlights changes made to the original version. You should upload this as a separate file labeled 'Revised Manuscript with Track Changes'.

* An unmarked version of your revised paper without tracked changes. You should upload this as a separate file labeled 'Manuscript'.

If you would like to make changes to your financial disclosure, competing interests statement, or data availability statement, please make these updates within the submission form at the time of resubmission. Guidelines for resubmitting your figure files are available below the reviewer comments at the end of this letter.

As the corresponding author, your ORCID iD is verified in the submission system and will appear in the published article. PLOS supports the use of ORCID, and we encourage all coauthors to register for an ORCID iD and use it as well. Please encourage your coauthors to verify their ORCID iD within the submission system before final acceptance, as unverified ORCID iDs will not appear in the published article. Only the individual author can complete the verification step; PLOS staff cannot verify ORCID iDs on behalf of authors.

We look forward to receiving your revised manuscript.

Kind regards,

Richard A. Bowen, DVM PhD

Academic Editor

PLOS Neglected Tropical Diseases

Sujatha Sunil

Section Editor

PLOS Neglected Tropical Diseases

Shaden Kamhawi

co-Editor-in-Chief

PLOS Neglected Tropical Diseases

orcid.org/0000-0003-4304-636XX

Paul Brindley

co-Editor-in-Chief

PLOS Neglected Tropical Diseases

orcid.org/0000-0003-1765-0002

**Additional Editor Comments (if provided):**

Your manuscript has been reviewed by 3 experts, each of whom has provided comments that need to be addressed. Please review the comments, edit your manuscript in response, and resubmit along with a point-by-point response to the reviewer comments and requests. Thank you.

**Journal Requirements:**

If the reviewer comments include a recommendation to cite specific previously published works, please review and evaluate these publications to determine whether they are relevant and should be cited. There is no requirement to cite these works unless the editor has indicated otherwise.

1) Please ensure that the CRediT author contributions listed for every co-author are completed accurately and in full.

At this stage, the following Authors/Authors require contributions: Lam Anh Nguyet, Nguyen To Anh, Du Tuan Quy, Nguyen Thi Thu Hong, Ngo Ngoc Quang Minh, Phung Nguyen The Nguyen, Pham Van Quang, Do Duong Kim Han, Nguyen Thi Han Ny, Le Nguyen Truc Nhu, Truong Hoang Chau Truc, Cao Thu Thuy, Nhung Ngoc Hoang, Tran Ba Thien, Le Kim Thanh, Nguyen Thanh Hung, Truong Huu Khanh, Cao Minh Hiep, Nghiem My Ngoc, Nguyen Thanh Dung, Le Nguyen Thanh Nhan, Nguyen Van Vinh Chau, Ha Manh Tuan, H. Rogier van Doorn, Marc Choisy, Tran Tan Thanh, and Le Van Tan. Please ensure that the full contributions of each author are acknowledged in the "Add/Edit/Remove Authors" section of our submission form.

The list of CRediT author contributions may be found here: https://journals.plos.org/plosntds/s/authorship#loc-author-contributions

2) Please provide an Author Summary. This should appear in your manuscript between the Abstract (if applicable) and the Introduction, and should be 150-200 words long. The aim should be to make your findings accessible to a wide audience that includes both scientists and non-scientists. Sample summaries can be found on our website under Submission Guidelines:

https://journals.plos.org/plosntds/s/submission-guidelines#loc-parts-of-a-submission

3) Please upload all main figures as separate Figure files in .tif or .eps format. For more information about how to convert and format your figure files please see our guidelines:

https://journals.plos.org/plosntds/s/figures

4) Please upload a copy of Figure Fig 1-5 which you refer to in your text on pages 10, 11, and 12. Or, if the figure is no longer to be included as part of the submission please remove all reference to it within the text.

5) We have noticed that you have uploaded Supporting Information files, but you have not included a list of legends. Please add a full list of legends for your Supporting Information files after the references list.

6) Please amend your detailed Financial Disclosure statement. This is published with the article. It must therefore be completed in full sentences and contain the exact wording you wish to be published.

i) State the initials, alongside each funding source, of each author to receive each grant. For example: "This work was supported by the National Institutes of Health (####### to AM; ###### to CJ) and the National Science Foundation (###### to AM)."

ii) State what role the funders took in the study. If the funders had no role in your study, please state: "The funders had no role in study design, data collection and analysis, decision to publish, or preparation of the manuscript."

iii) If any authors received a salary from any of your funders, please state which authors and which funders..

If you did not receive any funding for this study, please simply state: u201cThe authors received no specific funding for this work.u201d

7) We have amended your Competing Interest statement to comply with journal style. We kindly ask that you double check the statement and let us know if anything is incorrect.

8) Thank you for uploading your study's underlying data set. Unfortunately, the repository you have noted in your Data Availability statement does not qualify as an acceptable data repository according to PLOS's standards.

At this time, please upload the minimal data set necessary to replicate your study's findings to a stable, public repository (such as figshare or Dryad) and provide us with the relevant URLs, DOIs, or accession numbers that may be used to access these data. For a list of recommended repositories and additional information on PLOS standards for data deposition, please see

https://journals.plos.org/plosntds/s/recommended-repositories

**Reviewers' Comments:**

Reviewer's Responses to Questions

**Key Review Criteria Required for Acceptance?**

As you describe the new analyses required for acceptance, please consider the following:

**Methods**

-Are the objectives of the study clearly articulated with a clear testable hypothesis stated?

-Is the study design appropriate to address the stated objectives?

-Is the population clearly described and appropriate for the hypothesis being tested?

-Is the sample size sufficient to ensure adequate power to address the hypothesis being tested?

-Were correct statistical analysis used to support conclusions?

-Are there concerns about ethical or regulatory requirements being met?

Reviewer #1: no

Reviewer #2: The study design is appropriate, but the manuscript requires revisions to correct data inconsistencies, improve methodological transparency, and deepen the discussion of the study's limitations.

Reviewer #3: Minor revision. Please refer to Summary and General Comments.

**********

**Results**

-Does the analysis presented match the analysis plan?

-Are the results clearly and completely presented?

-Are the figures (Tables, Images) of sufficient quality for clarity?

Reviewer #1: no

Reviewer #2: The results are generally clearly presented, but several issues require correction. There are inconsistencies in sample numbers between the text and figures (e.g., EV-A71 positive vs. subtyped samples). Additionally, there are apparent typographical errors in the figure legends, such as incorrect years mentioned in Figure 4 and Figure 5, which need to be addressed.

Reviewer #3: Minor revision. Please refer to Summary and General Comments.

**********

**Conclusions**

-Are the conclusions supported by the data presented?

-Are the limitations of analysis clearly described?

-Do the authors discuss how these data can be helpful to advance our understanding of the topic under study?

-Is public health relevance addressed?

Reviewer #1: no

Reviewer #2: The conclusions are generally supported by the data, but the manuscript would be strengthened by a more in-depth discussion of the study's limitations, particularly regarding the potential bias of the hospital-based sample and the implications of the identified S17G mutation.

Reviewer #3: Minor revision. Please refer to Summary and General Comments.

**********

**Editorial and Data Presentation Modifications?**

Use this section for editorial suggestions as well as relatively minor modifications of existing data that would enhance clarity. If the only modifications needed are minor and/or editorial, you may wish to recommend “Minor Revision” or “Accept”.

Reviewer #1: (No Response)

Reviewer #2: Minor revision

Reviewer #3: None.

**********

**Summary and General Comments**

Use this section to provide overall comments, discuss strengths/weaknesses of the study, novelty, significance, general execution and scholarship. You may also include additional comments for the author, including concerns about dual publication, research ethics, or publication ethics. If requesting major revision, please articulate the new experiments that are needed.

Reviewer #1: It's a nice piece combining molecular epidemiology, phylogenetics, antigenic characterization and serosurveillance around an important outbreak, and the central narrative (single introduction of an antigenically unchanged B5 lineage into an immunologically naïve population) is compelling. However, there are several numerical inconsistencies between the text, figures, and legends that need to be resolved, plus some clarity and statistical issues.

1.Figure 4 legend appears to have the wrong year. The legend states the comparison is between "the 2023 emerging subgenogroup B5 and a subgenogroup B5 isolated in 2024," but the entire text, panel labels (B5/2014 vs B5/2023), and Methods (plasma from patients infected in 2014 and 2023) all describe a 2014-vs-2023 comparison, not 2024. Please correct "2024" to "2014" in the Figure 4 legend to avoid confusing readers about what is actually being compared.

2.Figure 5 x-axis/legend says "T4, Dec 2024," but the Methods and figure caption text state T4 = December 2023. This is a second instance of an apparent 2023/2024 mix-up and directly affects interpretation of the serosurveillance timeline, which is central to the paper's argument. Please correct the T4 label to "Dec 2023" in Figure 5 and double-check that no other 2023/2024 date errors exist elsewhere in the manuscript.

3.Minor percentage discrepancy for subgenogroup typing success. The Results text states subgenogroup determination was "successful in 231/299 (77.5%)," while Figure 1 reports 77.3% for the same numerator/denominator. 231/299 = 77.3%, so the text figure appears to be the error. Please correct "77.5%" to "77.3%" in the Results text to match Figure 1 and the underlying calculation.

4.Genogroup nomenclature inconsistency in the Introduction. The text states EV-A71 "is classified into eight genogroups, designated A to F," but A through F comprises only six letters/groups. Please verify the correct number of genogroups against the cited literature and correct either the number ("eight") or the letter range to be internally consistent.

5.Very small sample sizes underlying key seroprevalence estimates, without confidence intervals. Several headline percentages are based on extremely small denominators (e.g., 1/15, 1/10, 6/15), yet are reported only as point estimates (6.7%, 10%, 40%, etc.) without any measure of uncertainty. Given that a central claim of the paper is that seroprevalence "increased" over time and this is used to support a serosurveillance-based early-warning concept, the precision of these estimates matters a great deal. Please report 95% confidence intervals (e.g., Wilson or Clopper-Pearson) alongside all seroprevalence point estimates, and discuss the resulting uncertainty explicitly, especially for group 1 and group 2 where n is smallest.

6.Reference section header reads "REFEENCES", a typo for "REFERENCES." Please correct this heading.

7.Abstract: "0–<3 year-cold children" should read "year-old." Correct typo.

8.Methods (Data analysis section): "particiapnts" should read "participants." Correct typo.

9.Results: "with details availabe in three" should read "available." Correct typo.

Reviewer #2: The authors have conducted a comprehensive investigation into the 2023 EV-A71 outbreak in Vietnam, integrating molecular epidemiology, serology, and antigenic analysis. The study carries clear public health relevance. However, several points require further clarification and revision before the manuscript can be accepted.

1. Specific comments:

(1) In the Figure 4 legend, the authors refer to "a subgenogroup B5 isolated in 2024." Given that the study timeline spans 2022–2023, this date appears inconsistent and should be corrected to align with the actual isolation year.

(2) Similarly, the legend of Figure 5 contains an error indicating "Dec 2024," while the study period is defined as December 2022 to December 2023. This should be amended accordingly.

(3) The Results section reports that 360 patients were enrolled, of whom 299 tested positive for EV-A71, and subgenogroup data were available for 231 cases. The authors should clarify why the remaining 68 positive cases could not be subtyped and provide a more detailed discussion of this discrepancy.

(4) In the "Whole-genome sequence assembly" section, the authors describe using a reference-based mapping approach but do not specify the reference genome used. Please include the corresponding GenBank accession number(s) in this section.

(5) In Line 152, Geneious 8.1.should be Geneious v8.1.5.

(6) In the Data Availability section, I would recommend providing the NCBI SRA accession ID(s) rather than only the assembled sequences, to allow full transparency and reproducibility.

2. Questions on scientific logic and discussion

(1) The S17G mutation in VP1 N-terminus was shown not to affect immune escape, but its biological significance remains unclear. The authors should discuss whether this change might influence viral fitness or cell tropism, or whether it simply represents genetic drift.

(2) Serum samples were collected from hospital inpatients and may not fully represent community-wide pediatric immunity. This limitation should be acknowledged, along with its potential impact on population-level seroprevalence estimates.

(3) The proportion of severe cases (79.4%) is strikingly high. While hospital-based surveillance partly explains this, the authors should discuss whether mild cases in the community were systematically undercounted, and exercise caution when generalizing these findings to the broader outbreak profile.

Overall, the study is of interest, but I would recommend a minor revision to address the above issues.

Reviewer #3: This is a well-conducted study investigating the virological and epidemiological characteristics of the severe HFMD outbreak in Vietnam in 2023. Overall, the methodology is appropriate and sufficiently described. The study appears to have been conducted systematically, and the limitations have been adequately addressed in the Discussion.

However, there are several minor comments and queries that should be addressed to improve the clarity and accuracy of the manuscript:

1. Introduction

The authors state:

“Over the last decades, enterovirus A-71 (EV-A71) has emerged and become a major public health threat in the Western-Pacific region. Large outbreaks of EV-A71-associated hand, foot and mouth disease (HFMD) have occurred across the region, causing millions of hospitalizations in young children [1, 2].”

One of the references cited is relatively old (1998), while the other is a review article. The authors should reconsider the references used to support this statement and, where possible, include more recent epidemiological studies or surveillance data to provide up-to-date evidence for the continuing public health significance of EV-A71 in the Western-Pacific region.

2. Introduction

The authors state:

“…and most recently in 2023 (>180,000 cases and 31 deaths) [9].”

However, Reference 9 appears to report 7 deaths, rather than 31 deaths. Please verify this information and revise the statement accordingly. If the figure of 31 deaths is supported by another reliable source, the authors should provide an appropriate reference.

3. Materials and Methods – Serum Bank and Sample Selection for Serosurveillance

The description of the serum sample selection is unclear:

“4 time points of collection × 15 age classes × 5 samples”

It is not clear whether the study included 15 age classes with 5 samples collected at each of 4 time points, or 16 age (0 to 15 age) classes with 4 samples per age class. Please clarify the sampling scheme and provide the total number of serum samples included in the serosurveillance analysis.

4. Materials and Methods – Enterovirus Diagnostics and Serotype Determination

The authors state that viral RNA was extracted from rectal swabs collected from study participants. Please provide a justification for using rectal swabs rather than throat swabs for viral RNA detection.

Previous work has suggested that throat swabs may provide better diagnostic sensitivity than rectal swabs for EV-A71 detection. For example:

Ooi EE, et al. Evaluation of Different Clinical Sample Types in Diagnosis of Human Enterovirus 71-Associated Hand-Foot-and-Mouth Disease. Journal of Clinical Microbiology. 2007;45(6). https://doi.org/10.1128/JCM.01394-06.

Please discuss the rationale for the choice of specimen type and whether the use of rectal swabs could have influenced the detection rate of EV-A71 or other enteroviruses.

In addition, it is notable that CV-A16 was not detected in this study. Please provide some discussion regarding this observation. For example, could this finding be related to the sampling population, specimen type, circulating enterovirus epidemiology, or the timing of sample collection?

5. Discussion

The authors conclude:

“EV-A71 infection represents an ongoing public health threat, currently affecting countries in the Asia-Pacific region, but with a high potential to become a global concern, and therefore should be closely monitored.”

The authors may wish to broaden this statement to acknowledge that other enteroviruses associated with HFMD, particularly CV-A6 and CV-A10, are also increasingly important from a public health perspective. These viruses have been associated with outbreaks and, in some settings, severe or atypical clinical manifestations.

Therefore, the authors could consider emphasizing the importance of continued surveillance of EV-A71 as well as other HFMD-associated enteroviruses, rather than focusing exclusively on EV-A71. This would provide a broader perspective on the evolving epidemiology and public health significance of HFMD.

**********

PLOS authors have the option to publish the peer review history of their article (what does this mean?). If published, this will include your full peer review and any attached files.

If you choose “no”, your identity will remain anonymous but your review may still be made public.

**Do you want your identity to be public for this peer review?**  For information about this choice, including consent withdrawal, please see our Privacy Policy.

Reviewer #1: **Yes:**  Ahmed Hamdy Ghonaim

Reviewer #2: No

Reviewer #3: No

[NOTE: If reviewer comments were submitted as an attachment file, they will be attached to this email and accessible via the submission site. Please log into your account, locate the manuscript record, and check for the action link "View Attachments". If this link does not appear, there are no attachment files.]

**Figure resubmission:**

**Reproducibility:**

To enhance the reproducibility of your results, we recommend that authors of applicable studies deposit laboratory protocols in protocols.io, where a protocol can be assigned its own identifier (DOI) such that it can be cited independently in the future. Additionally, PLOS ONE offers an option to publish peer-reviewed clinical study protocols. Read more information on sharing protocols at https://plos.org/protocols?utm_medium=editorial-email&utm_source=authorletters&utm_campaign=protocols

# Author comment: Author response to Decision Letter 1

**DOI:** 10.1371/journal.pntd.0014789.r002

9 Sep 2026

Attachment Submitted filename: EV-A71 2023 outbreak Reviewers comments R1.docx

# Editor report: Decision Letter 1

**DOI:** 10.1371/journal.pntd.0014789.r003

**Authors:** Richard Bowen, Sujatha Sunil

20 Sep 2026

Dear Dr Tan,

We are pleased to inform you that your manuscript 'Virological and epidemiological investigations of a severe hand foot and mouth disease outbreak, Vietnam, 2023' has been provisionally accepted for publication in PLOS Neglected Tropical Diseases.

Before your manuscript can be formally accepted you will need to complete some formatting changes, which you will receive in a follow up email. A member of our team will be in touch with a set of requests.

Please note that your manuscript will not be scheduled for publication until you have made the required changes, so a swift response is appreciated.

IMPORTANT: The editorial review process is now complete. PLOS will only permit corrections to spelling, formatting or significant scientific errors from this point onwards. Requests for major changes, or any which affect the scientific understanding of your work, will cause delays to the publication date of your manuscript.

Should you, your institution's press office or the journal office choose to press release your paper, you will automatically be opted out of early publication. We ask that you notify us now if you or your institution is planning to press release the article. All press must be co-ordinated with PLOS.

Thank you again for supporting Open Access publishing; we are looking forward to publishing your work in PLOS Neglected Tropical Diseases.

Best regards,

Richard A. Bowen, DVM PhD

Academic Editor

PLOS Neglected Tropical Diseases

Sujatha Sunil

Section Editor

PLOS Neglected Tropical Diseases

Shaden Kamhawi

co-Editor-in-Chief

PLOS Neglected Tropical Diseases

orcid.org/0000-0003-4304-636XX

Paul Brindley

co-Editor-in-Chief

PLOS Neglected Tropical Diseases

orcid.org/0000-0003-1765-0002

***********************************************************

Thank you for the careful and extensive editing of your manuscript. I consider this a valuable contribution to the field and find it now acceptable for publication.

p.p1 {margin: 0.0px 0.0px 0.0px 0.0px; line-height: 16.0px; font: 14.0px Arial; color: #323333; -webkit-text-stroke: #323333}span.s1 {font-kerning: none

# Editor report: Acceptance letter

**DOI:** 10.1371/journal.pntd.0014789.r004

**Authors:** Richard Bowen, Sujatha Sunil

Dear Dr Tan,

We are delighted to inform you that your manuscript, "Virological and epidemiological investigations of a severe hand foot and mouth disease outbreak, Vietnam, 2023," has been formally accepted for publication in PLOS Neglected Tropical Diseases.

We have now passed your article onto the PLOS Production Department who will complete the rest of the publication process. All authors will receive a confirmation email upon publication.

The corresponding author will soon be receiving a typeset proof for review, to ensure errors have not been introduced during production. Please review the PDF proof of your manuscript carefully, as this is the last chance to correct any scientific or type-setting errors. Please note that major changes, or those which affect the scientific understanding of the work, will likely cause delays to the publication date of your manuscript. Note: Proofs for Front Matter articles (Editorial, Viewpoint, Symposium, Review, etc...) are generated on a different schedule and may not be made available as quickly.

Soon after your final files are uploaded, the early version of your manuscript will be published online unless you opted out of this process. The date of the early version will be your article's publication date. The final article will be published to the same URL, and all versions of the paper will be accessible to readers.

For Research Articles, you will receive an invoice from PLOS for your publication fee after your manuscript has reached the completed accept phase. If you receive an email requesting payment before acceptance or for any other service, this may be a phishing scheme. Learn how to identify phishing emails and protect your accounts at https://explore.plos.org/phishing.

Thank you again for supporting open-access publishing; we are looking forward to publishing your work in PLOS Neglected Tropical Diseases.

Best regards,

Shaden Kamhawi

co-Editor-in-Chief

PLOS Neglected Tropical Diseases

Paul Brindley

co-Editor-in-Chief

PLOS Neglected Tropical Diseases
